# Elucidating temporal resource allocation and diurnal dynamics in phototrophic metabolism using conditional FBA

**DOI:** 10.1038/srep15247

**Published:** 2015-10-26

**Authors:** Marco Rügen, Alexander Bockmayr, Ralf Steuer

**Affiliations:** 1 Humboldt-Universität zu Berlin, Institut für Theoretische Biologie (ITB), Invalidenstr. 43, D-10115 Berlin, Germany; 2 Freie Universität Berlin, Research Center Matheon, FB Mathematik und Informatik, Arnimallee 6, D-14195 Berlin, Germany

## Abstract

The computational analysis of phototrophic growth using constraint-based optimization requires to go beyond current time-invariant implementations of flux-balance analysis (FBA). Phototrophic organisms, such as cyanobacteria, rely on harvesting the sun’s energy for the conversion of atmospheric CO2 into organic carbon, hence their metabolism follows a strongly diurnal lifestyle. We describe the growth of cyanobacteria in a periodic environment using a new method called conditional FBA. Our approach enables us to incorporate the temporal organization and conditional dependencies into a constraint-based description of phototrophic metabolism. Specifically, we take into account that cellular processes require resources that are themselves products of metabolism. Phototrophic growth can therefore be formulated as a time-dependent linear optimization problem, such that optimal growth requires a differential allocation of resources during different times of the day. Conditional FBA then allows us to simulate phototrophic growth of an average cell in an environment with varying light intensity, resulting in dynamic time-courses for all involved reaction fluxes, as well as changes in biomass composition over a diurnal cycle. Our results are in good agreement with several known facts about the temporal organization of phototrophic growth and have implications for further analysis of resource allocation problems in phototrophic metabolism.

Constraint-based modeling of cellular metabolism, most notably flux-balance analysis (FBA), has become a versatile tool to study the functioning and optimality of large-scale metabolic networks^1^,^2^. A major prerequisite for the application of FBA, however, is the assumption of a time-invariant metabolism: For each internal compound within the reconstructed network, the sum of producing fluxes must equal the sum of consuming fluxes, thereby ensuring mass-conservation of the respective compounds. The assumption of a time-invariant mass balance imposes significant constraints on the feasible flux space, and is one of the pillars for the explanatory and predictive success of FBA.

The assumption of a time-invariant metabolism, however, does not always hold. Almost all organisms are known to exhibit temporal variations in their metabolism, for example in response to environmental conditions, such as nutrient availability or diurnal rhythms, or imposed by different cellular requirements during cellular growth and division.

In particular phototrophic organisms, whose metabolism is based on harvesting the sun’s energy, typically exhibit a diurnal lifestyle. Phototrophic metabolism involves at least two distinct phases: a light phase that includes synthesis of cellular precursors as well as storage compounds, and a subsequent dark phase where storage compounds are mobilized to ensure survival in the absence of light. Typically, the transition between these two states is not abrupt and phototrophic metabolism is characterized by continuous temporal change.

As yet, such temporal transitions are difficult to describe using conventional FBA. While there have been a number of recent metabolic reconstructions for phototrophic organisms, including reconstructions for cyanobacteria^3^,^4^,^5^,^6^,^7^, algae^8^,^9^,^10^,^11^,^12^ and higher plants^13^,^14^,^15^,^16^, constraint-based modeling of the respective metabolic networks is often either confined to a constant light environment or heterotrophic growth on extracellular carbon sources. Only a few recent studies take different phases of light availability into account, such as Knoop *et al.*^7^, who simulated fluxes over a full diurnal cycle, Muthuraj *et al.*^17^, who use dynamic FBA (dFBA) to capture light-dark metabolism over discretized time intervals, and Cheung *et al.*^18^ who describe a flux balance model that captures interactions between light and dark metabolism in C3 and CAM leaves. In this work, we seek to investigate the temporal changes in phototrophic metabolism over a full diurnal cycle using a new method called conditional FBA (cFBA). Our computational approach is based on the notion that metabolism is inherently autocatalytic. For example, to synthesize cellular building blocks, such as amino acids, requires the action of enzymes, which are themselves translated from amino acids by ribosomes. Ribosomes are synthesized from RNA and amino acids. Likewise, phototrophic CO_2_ fixation requires energy and redox equivalents in the form of ATP and NADPH, derived from the photosynthetic light reactions. Light harvesting makes use of pigments that are products of metabolism. Clearly, such autocatalytic cycles give rise to multiple interdependencies within cellular metabolism. The temporal order in which the cellular building blocks are synthesized itself influences the metabolic capacity at subsequent timepoints. It can be assumed, therefore, that the cellular machinery has evolved a suitable regulatory structure to ensure an appropriate, if not optimal, temporal allocation of its resources. We note that the assumption of such optimally adapted allocation of cellular resources is commonly employed in conventional FBA. Furthermore, the fact that cellular growth and resource allocation, at least to a certain extent, can be understood based on the assumption of optimal resource allocation, has been demonstrated experimentally^19^,^20^. At the core of our constraint-based metabolic optimization problem is therefore the objective to maximize biomass synthesis over a full diurnal cycle, constrained by the conditional interdependencies of cellular metabolism. From a computational perspective, our approach is a synthesis of dynamic FBA^21^ that allows to incorporate temporal changes in substrate concentrations, and Resource Balance Analysis (RBA) that incorporates the constraints on resource allocation into FBA^22^. Conditional FBA can also be related to ME (metabolism and macromolecular expression) models^23^, as well as to dynamic enzyme-cost FBA (deFBA)^24^, which provides a dynamic programming framework for metabolic networks coupled to gene expression. Our focus, however, is phototrophic growth of the cyanobacterium *Synechocystis* sp. PCC 6803 over a full diurnal cycle. Our ultimate aim is to understand the principles of temporal resource allocation for phototrophic growth in a periodic environment.

## Results

### Conditional dependencies in metabolism

Our approach is based on the fact that cellular growth is inherently autocatalytic. We implement the corresponding constraint-based optimization problem by constraining each flux by the amount or activity of the compound that facilitates the respective flux. In particular, we assume that the flux through any metabolic reaction is constrained by the amount of the respective enzyme, the total synthesis rate of enzymes is constrained by the amount of ribosomes, and light harvesting is constrained by the amount of pigments. Importantly, each of these compounds is a product of metabolism itself. To simulate time-dependent metabolism, time is subdivided into discrete intervals, such that fluxes at different time intervals are distinct entities. Intracellular compounds are allowed to accumulate and the difference between synthesis and consumption rates over time corresponds to an increase or decrease of the respective amounts. The total amount of accumulated compounds can itself be subject to minimization to reflect cellular size constraints. The network can adopt any flux distribution that is consistent with the specified constraints. A flux distribution is obtained by maximizing a given objective function. Suitable objective functions are the accumulated concentration of a target compound over the entire simulation period, or, in our case, maximal growth of the compound amount vector over a full diurnal period.

### A simple example

**Figure 1:**
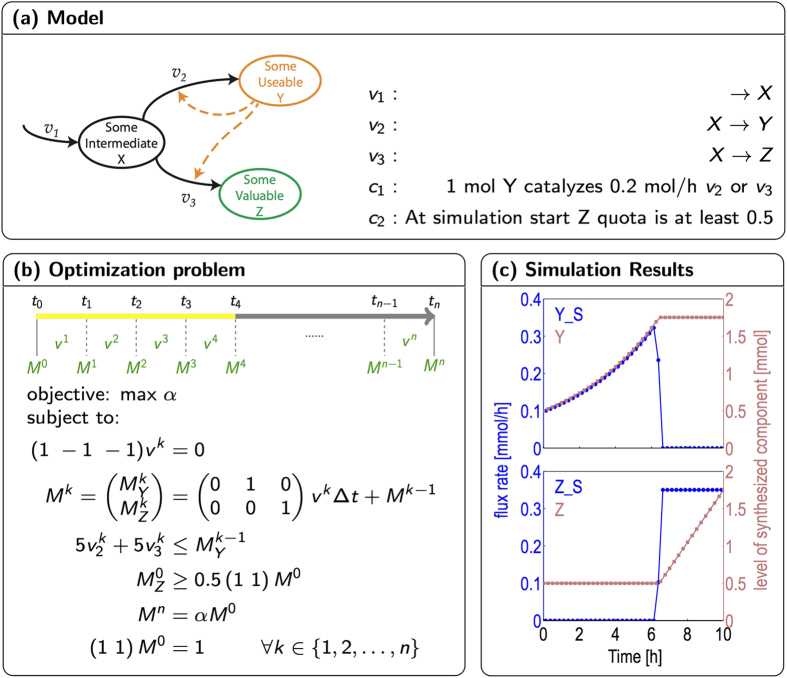
Conditional FBA illustrated using a simple example. The network consists of only three reactions, such that the compound *X* is taken up and either converted into a target compound *Z*, or into an enzyme *Y* that facilitates the formation of *Z* as well as of itself. (**a**) The model and its associated reactions. (**b**) The discretization of time and the corresponding optimization problem. *v*^*k*^ is the flux distribution in *k*-th time interval, 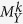 and 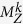 are the compound amounts of Y and Z at time point *t*_*k*_. (**c**) The output of the constraint optimization problem. Shown are the synthesis rates (blue) (*Y*_*S*, *Z*_*S*) and the accumulated amounts (red) of the compounds *Y* and *Z*. The objective is to maximize growth, that is, to increase the compound amount vector *M*^0^ by a factor *α*, with the additional constraint that the relative quota of *Z* must be at least 0.5 at simulation start. The displayed solution is unique.

Our approach is illustrated in Fig. 1. As a simple example, we consider a model that consists of only three cellular compounds *X*, *Y*, and *Z*. We assume that the compound *X* can be taken up with a rate *ν*_1_ and its conversion into the final product *Z* is catalyzed by the enzyme *Y*. Hence the respective conversion flux *ν*_3_ is bounded by the availability of *Y*. The enzyme *Y* is itself synthesized from *X*, the respective reaction *ν*_2_ is again catalyzed by *Y*. Synthesis of *Z* can then be cast as an optimization problem, such that the compound *X* is balanced whereas the compounds *Y* and *Z* are allowed to accumulate. The corresponding global optimization problem can be described by a set of linear constraints, given in Fig. 1b. We then consider the periodic optimization problem, such that the final vector of compound amounts *M*(*t*_end_) is a multiple of the initial compound amounts *M*(*t*_start_),


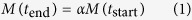


where *T* = *t*_end_ − *t*_start_ denotes the time interval, typically a full diurnal period. The optimization objective is to maximize the factor *α*. Importantly, we do not aim to pre-assign the detailed composition of the compound amount vector *M*. Rather, the cellular composition emerges as a result of the optimization problem. Only for compounds without immediate catalytic activity, the optimization problem must be supplemented with additional constraints on minimal compound quotas to ensure their production within the simulation period. In the example shown in Fig. 1, the minimal quota of the compound *Z* is 0.5. The quota is enforced only at a single timepoint (simulation start) and the relative amount may change during the simulation.

The results of the time-dependent optimization problem are shown in Fig. 1c. At the start of the simulation, only the compound *Y* is synthesized. At a time *t* ≈ 6.2 *h*, the system undergoes a metabolic switch. At this point, the system starts to synthesize the target compound *Z* which is subsequently accumulated linearly during the remaining simulation period. In the following, we employ this approach to simulate cyanobacterial growth in a periodic environment. Definitions and computational implementations are detailed in the section “Methods”. Time courses of compound synthesis are reported in absolute amounts, unless otherwise noted, not in concentrations relative to total biomass. We note that, at this point, we neglect potential influences of cell division and only consider diurnal cellular growth. The computational results can therefore be interpreted to represent an average cell in a growing culture under diurnal illumination.

### A minimal model of phototrophic growth

**Figure 2:**
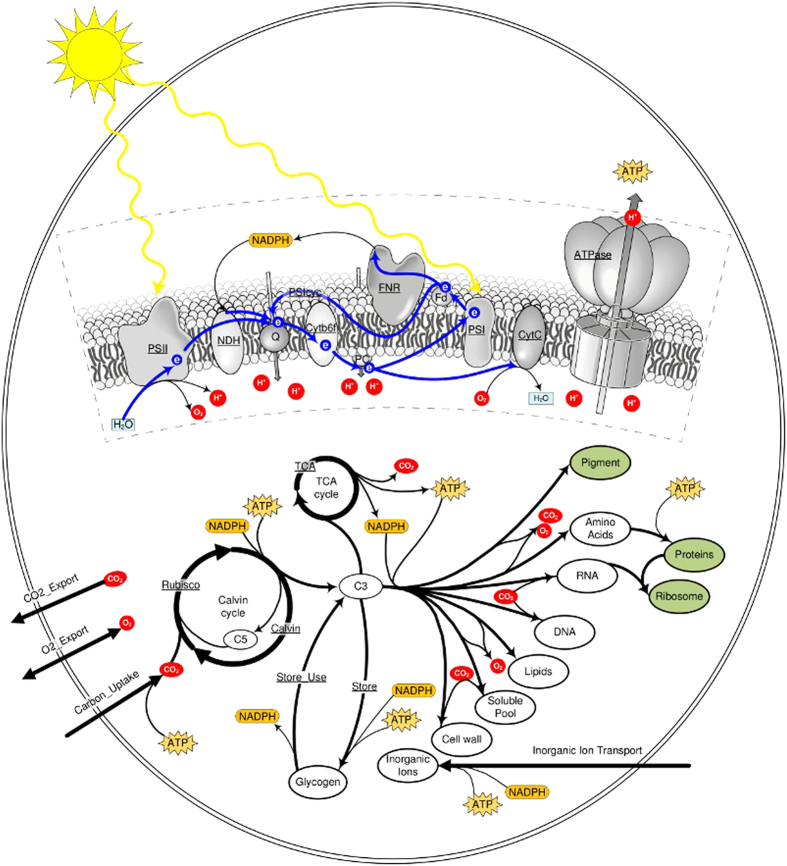
A model of *Synechocystis* sp. PCC 6803. Underlined labels indicate reactions, framed oval elements specify cellular compounds. Compounds in bold frame can accumulate during the simulation period. The compound Protein represents the full set of enzymes required to catalyze the metabolic reactions in the system. See Table 1 for a full list of reactions.

To study phototrophic growth over a full diurnal cycle, we implement a simplified metabolic network model of the cyanobacterium *Synechocystis* sp. PCC 6803, based on an available genome-scale reconstruction of the organism^7^. The model incorporates the photosynthetic light reactions, linear and cyclic electron transport, CO_2_ uptake, carbon fixation by the Calvin-Benson (CB) cycle, accumulation of glycogen as a storage compound, uptake of inorganic ions, the synthesis of precursors for cellular growth, as well as a TCA cycle and respiration to account for storage-based metabolism in the absence of light. The model, depicted in Fig. 2, consists of 52 reactions and 50 compounds. In brief, photons are harvested in the antenna complexes associated to photosystem II, resulting in water splitting, the release of O_2_ and reduction of plastoquinone (QH2). Transport of the electron down the electron transport chain (ETC) results in an accumulation of protons in the thylakoid lumen and generation of NADPH via photosystem I (PSI) and the FNR complex. Cyclic electron transfer is encoded as a distinct process mediated by PSI. We note that the molecular details of cyclic electron transport are as yet not fully understood. ATP is regenerated by the ATPase complex. The ETC also incorporates the enzyme complex NDH and cytochrome C oxidase to allow for respiratory activity in the absence of light.

In the cytosol, inorganic carbon is taken up and incorporated into organic compounds. With respect to carbon uptake, no distinction is made between CO_2_ and bicarbonate 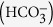. The CB cycle is represented by a single reaction that yields two molecules of the 3-carbon molecule 3-phosphoglycerate (C3). The molecule C3 represents the central metabolic precursor for all subsequent biosynthesis reactions. Biosynthesis is described by overall reactions for pigments, amino acids, RNA, DNA, lipids, soluble metabolites, and cell wall. The respective stoichiometries, including consumption of ATP and NADPH, are derived from the available genome-scale reconstruction^7^. Amino acids and RNA are further converted into ribosomes and proteins. In addition to biosynthesis, the central precursor molecule C3 can be converted into the storage compound glycogen, or channeled into the TCA cycle to derive NADPH that is fed into the respiratory ETC. For simplicity, no distinction is made between NADH and NADPH. A full list of reactions is given in Table 1. Details of model construction are given in the section “Methods”.

### Implementing conditional dependencies

Growth of the system is constrained by multiple interdependencies. The respective constraints are incorporated as inequalities of the form


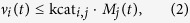


where *M*_*j*_(*t*) denotes the amount of the cellular component *j* that constrains flux *ν*_*i*_(*t*) through the reaction *i* at time *t*. The factors kcat_*i*,*j*_ denote catalytic efficiencies. The key constraints incorporated within the model are: (i) light harvesting in photosystem I (PSI) and II (PSII) is constrained by the amount of available pigments; (ii) the total enzyme synthesis rate is constrained by the amount of available ribosomes; (iii) each metabolic reaction is associated with a dedicated enzyme, whose amount limits the flux through the respective reaction. In addition, the model incorporates several constraints on relative compound quotas. In particular, cellular components that do not fulfill an immediate catalytic role, such as cell wall, inorganic ions, soluble metabolites, and lipids must be present in minimal quotas relative to cellular biomass, where biomass is a weighted sum of all cellular components. The quotas ensure that compounds without catalytic activity are synthesized. For simplicity, a subset of metabolites in central metabolism is assumed to be mass-balanced, i.e., these metabolic intermediates are not required to accumulate prior to cell division. We note that this approximation is also employed in conventional FBA, where dilution of internal metabolites is commonly neglected despite the synthesis of biomass (cell growth). The approximation can be straightforwardly eliminated, such that all cellular compounds are part of the compound amount vector *M*, at the expense of additional computational effort. Here, the approximation is motivated by the fact that the metabolic burden due to dilution of internal metabolites is small compared to the turnover of metabolic flux for the synthesis of main cellular components.

A full list of dependencies is provided in Table 2. The catalytic efficiencies of compounds are estimated based on average cellular composition, as reported in the genome-scale reconstruction, assuming self-consistency of the FBA flux solution. That is, the known cellular fraction of compounds in the reported biomass function is assumed to be sufficient to replicate the cell given a light intensity for which doubling time is approximately 24 h. See section “Methods” for details.

### A day in the life of Synechocystis 6803

Using the model of *Synechocystis* sp. PCC 6803 shown in Fig. 2, phototrophic growth is simulated over a full diurnal cycle. The objective is to maximize the factor *α* in Equation (1) over a period of *T* = 24 h, corresponding to maximal overall growth of an average cell. To this end, the day is subdivided into two distinct phases, a light phase of *T*_*L*_ = 12 h and a subsequent dark phase of *T*_*D*_ = 12 h. During the light phase, light utilization is only limited by the amount of pigments. Time is discretized into *n* = 48 uniform intervals. See section “Methods” for the choice and impact of different discretization intervals.

**Figure 3:**
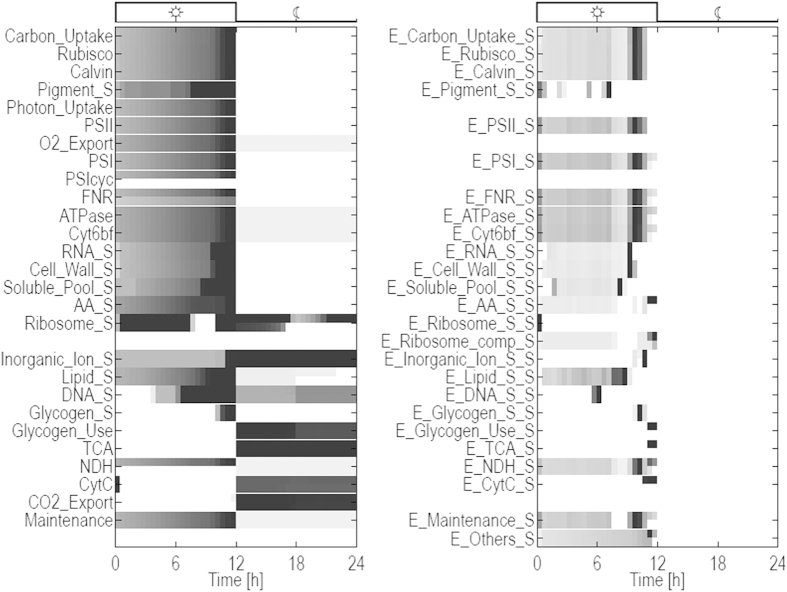
Flux rates over a full diurnal cycle. A prefix 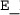 denotes compounds that catalyze reactions. A 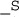 denotes the synthesis flux of the respective biomass compounds. For example, the term 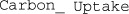 denotes the flux through the carbon uptake reaction, while 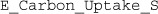 denotes the rate at which the transporter is synthesized. Flux rates are normalized such that the color 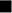 indicates the maximum value. White color indicates no flux activity. Each rate is represented by two rows. The upper row encodes the upper bounds of the normalized flux rates, the lower rows encode lower bounds. In the absence of flux variability, both rows coincide. The flux 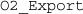 switches sign during the cycle, with negative rates during night (net O_2_ uptake). Shown is the absolute value.

**Figure 4:**
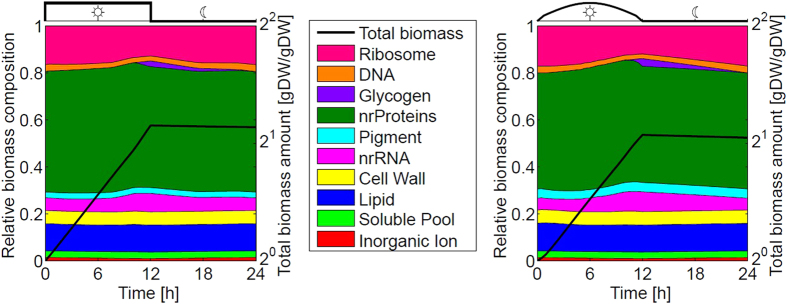
Relative biomass composition over a full diurnal cycle. The left panel shows the results for the scenario where light harvesting during day is constrained by the availability of pigments only. The right panel shows the results for a bell-shaped curve of light intensity. Total biomass over the full diurnal period is shown in logarithmic coordinates.

Figure 3 shows the temporal changes in all 52 resulting reaction fluxes over the entire diurnal period. The compound composition vector *M* at dawn is itself part of the optimization problem. That is, the relative quotas of enzymes, ribosomes, pigments and glycogen are not constrained and emerge as a result of the optimization. Minimal relative quotas are set for the compounds cell wall, lipids, RNA, inorganic ions, and soluble metabolites, as described above. The compound DNA must fulfill a minimal quota only at the beginning and, implicitly, at the end of the simulation period. Solving the constrained optimization problem over a full diurnal cycle, we obtain the time-evolution of each flux within the system, such that the solution ensures an optimal resource allocation for cellular growth. That is, at each time point the local fluxes are organized such that the factor *α* in Equation (1) is maximal over the full time interval *T* = 24 *h*. We note that the solution is not necessarily unique and flux variability analysis is performed at each time point. To this end, the maximal factor *α* is fixed and all fluxes and compound amounts are subsequently minimized and maximized in each time step, corresponding to two optimization runs per compound and flux per time step. The resulting flux variability is indicated in all subsequent figures. Overall growth and relative biomass composition over the full diurnal cycle is shown in Fig. 4. Detailed time courses of all cellular compounds are provided in the Supplementary Information.

The simulation results recover several known features of cyanobacterial growth. As expected, most compounds, as well as total biomass, exhibit exponential growth during the light period. Carbon fixation and flux through the CB cycle is exponentially increasing during the day. Also other synthesis fluxes, such as for cell wall and RNA exhibit exponential increase during the day. The storage compound glycogen is synthesized during late afternoon (
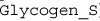
). Reactions associated with cellular respiration (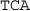, 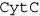) are not active during the light period, only 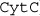 exhibits a brief activity in the early morning. The synthesis reactions of enzymes (denoted with the suffix 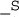) only show activities during the light phase. Most enzyme synthesis reactions exhibit more pronounced temporal patterns and peak before sundown.

Several reactions that synthesize catalyzing compounds (enzymes) are confined to brief periods, most notably the production of the enzymes responsible for the synthesis of ribosomes, DNA, glycogen usage, and import of inorganic ions. Intermittent behaviour is also observed for several reactions fluxes, most notably 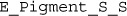. To ensure that the observed patterns indeed reflect the optimized behaviour for these synthesis rates, the solution has been tested for possible numerical instabilities (see section “Methods” for details). Overall, flux variability analysis reveals only minor variability in the reaction rates. We note that highly intermittent synthesis rates mostly correspond to the synthesis of enzymes. These rates are not directly dependent on environmental parameters, such as light, and are only constrained by the global ribosomal capacity and availability of precursors.

### Night metabolism and utilization of storage compounds

At dusk, the system undergoes a pronounced metabolic shift. Night metabolism is dominated by utilization of glycogen that is metabolized via the TCA cycle. The respiratory reactions provide ATP to meet the requirements for maintenance metabolism. A small number of other synthesis reactions remain active in the absence of light, such as the production of DNA, ribosomes, lipids, as well as uptake of inorganic ions. We note, however, that the total night synthesis of lipids makes up less than 2% of the total lipid production over the full simulation period. The time courses for all cellular compounds and synthesis fluxes are provided in the Supplementary Information. We observe that, during the light period, all cellular compounds operate at maximal capacity as long as light is available. That is, the observed flux corresponds to the constraint implemented in Equation (2). See Supplementary Information for a detailed account of capacity usage.

### Growth under varying light

**Figure 5:**
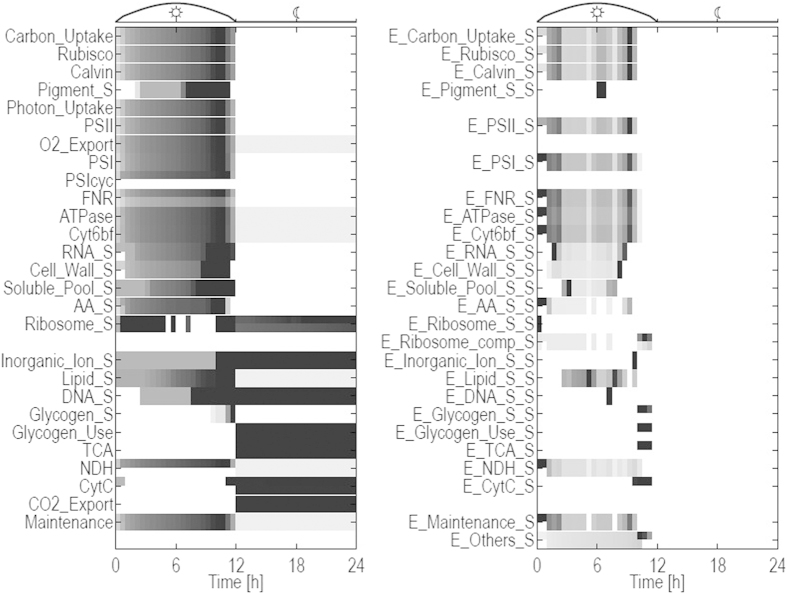
Flux rates over a full diurnal cycle using a bell shaped light curve. A prefix 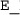 denotes compounds that catalyze reactions. A suffix 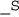 denotes the synthesis flux of the respective biomass compound. Flux rates are normalized such that the color 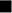 indicates the maximum. White color indicates no flux activity. Each rate is represented by two rows. The upper row encodes the upper bounds of the normalized flux rates, the lower rows encode lower bounds. In the absence of flux variability, both rows coincide. The flux 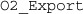 switches sign during the cycle, with negative rates during night (net O_2_ uptake). Shown is the absolute value.

As yet, light availability was considered to be a binary variable and light absorption was only limited by the amount of available pigments. However, in most natural environments the intensity of sunlight follows an approximately bell-shaped curve and light harvesting is a function of both, light availability and the amount of pigments. Extending the optimization problem considered above, we therefore implement a bell-shaped light intensity. Simulations were carried out as described above, such that light absorption is proportional to the amount of pigment multiplied with the time-dependent light intensity. Figure 5 shows all 52 reaction fluxes over the entire diurnal period, analogous to Fig. 3. Individual time courses are provided in the Supplementary Information. Qualitatively, the time-dependent flux changes are similar as for constant light availability, albeit with some notable differences. In particular, most synthesis fluxes now peak before dusk, corresponding to the diminishing light availability at the end of the day.

Small differences are also observed in the overall biomass composition over the full simulation period, shown in Fig. 4 (right panel). More pronounced changes are observed in the degree of capacity utilization of reactions. Specifically, for a bell-shaped light intensity most enzymes operate below their maximal capacity during dawn and dusk. Only a few hours after dawn, the reaction fluxes reach the maximal capacity given by the amount of their respective catalyzing compound, see Supplementary Information.

### Robustness and the limits of growth

**Figure 6:**
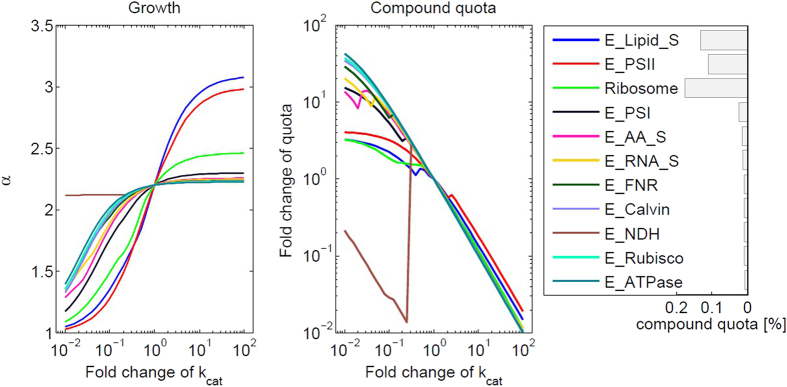
Dependency of overall growth factor *α* and of the compound composition vector *M* on the catalytic activities kcat_*i,j*_. The bars in the legend (rightmost column) indicate the relative amount of compounds, as determined by the compound composition vector *M* after the full diurnal period. Only the top 11 compounds are shown. (**A**) Increasing kcat_*i,j*_ typically results in higher *α*. (**B**) Increasing kcat_*i,j*_ typically results is a decreasing amount of the respective compound in the compound composition vector. A change in kcat of E_NDH induces a discontinuous transition. See text for details.

While the overall simulation results are in good agreement with several known features of diurnal phototrophic metabolism, it must be expected that the precise forms of the time courses also depend on parameter values and model assumptions. In particular the catalytic efficiencies kcat_*i*,*j*_, while being reasonable first approximations, are not precise estimates of the actual values. We therefore study the robustness of our results with respect to these values. Specifically, we evaluate the respective change in maximal growth yield, corresponding to the factor *α*, in response to a change in the catalytic efficiencies kcat_*i*,*j*_ of each catalyzing compound. The catalytic efficiencies are perturbed individually for each compound, with a fold change 10^−3^ to 10^+3^ from the reference value, and the effect on the factor *α* (growth), as well as on the compound composition vector *M*, is recorded. In addition, we record if a given synthesis reaction is active in the absence of light. Results are shown in Fig. 6 for the case of binary light availability. As expected, an increase in any of the kcat_*i*,*j*_, corresponding to a catalytic compound with higher catalytic activity, results in an increase of the factor *α*, hence higher growth yield over one diurnal period. The effect is most pronounced for compounds that make a significant contribution to the compound composition vector *M*, such as photosystem II and ribosomes. Similar, an increase in any of the kcat_*i*,*j*_ results in a reduction of the amount of the respective compound in the compound composition vector *M*. With respect to the results shown in Fig. 6, we note that only ribosomes and PSII correspond to actual cellular compounds, whereas other compounds, such as 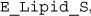, represent a conglomerate of enzymes associated to a specific pathway, such as lipid synthesis. The respective kcat_*i*,*j*_ therefore correspond to a global change in the respective enzyme properties.

Differing from the behaviour of most other compounds, we observe a sudden discontinuous decrease of 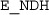, a compound in the electron transport chain, upon a decrease in its kcat_*i*,*j*_. Given our model definitions, 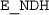 is involved as a capacity limiting compound in cyclic electron flow and hence ATP generation during day. With decreasing catalytic efficiency of 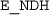, more compound is needed to establish the required flux, hence the costs of establishing the pathway increases. Above a certain threshold, the system undergoes a switch and an alternative pathway using 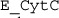 is utilized. The synthesis of 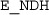 nonetheless remains essential for the use of NADPH in the absence of light, hence a further decrease in its kcat_*i*,*j*_ results again in an increasing amount of 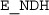 to meet the requirements for night metabolism. The switch is therefore an outcome of an interplay between different synthesis costs per catalytic rate of alternative pathways.

**Figure 7:**
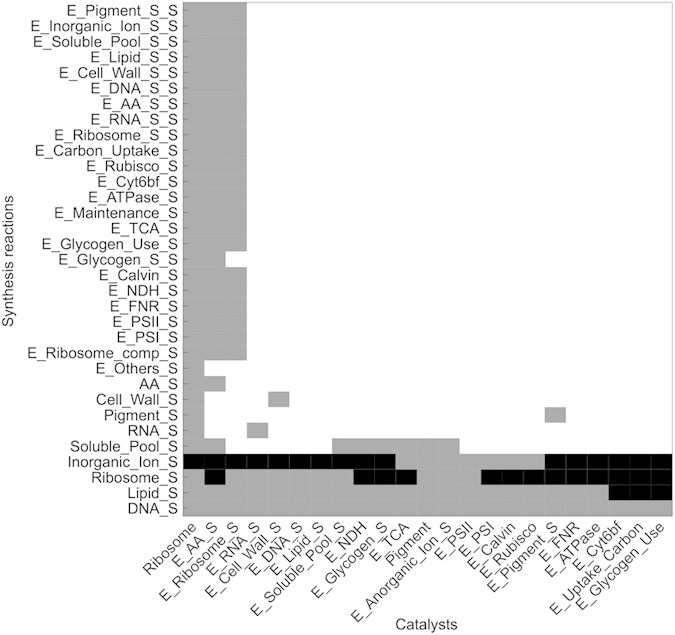
Activities of cellular synthesis reactions in the absence of light as a function of catalytic efficiencies. Black squares indicate that the respective synthesis reaction is also active in the absence of light, irrespective of the value of *k*_*cat*_ (varied with fold change 10^−3^ to 10^+3^ from the reference value). Gray squares indicate that the respective synthesis reaction is also active in the absence of light for some, but not all, values of *k*_*cat*_ within the considered interval (fold change 10^−3^ to 10^+3^ from the reference value). Specifically the uptake of inorganic ions, as well as the synthesis of ribosomes, lipids and DNA exhibits activity in the absence of light.

A related question concerns the activity of cellular metabolism during the night. Metabolism in the absence of light is dominated by the (enforced) requirement for ATP for cellular maintenance. Nonetheless, as shown in Figs 3 and 5, several cellular compounds are also synthesized in the absence of light. It is known that *Synechocystis* sp. PCC 6803 exhibits a highly reduced metabolism during the night, and no growth is observed. Nonetheless synthesis of few cellular compounds may proceed also during the night phase. We therefore investigate the possibility of synthesis reactions in the absence of light in the dependence of the catalytic efficiencies kcat_*i*,*j*_. For each catalyst the value of kcat_*i*,*j*_ was varied around its estimated reference value (fold change 10^−3^ to 10^+3^) and the activity of synthesis reactions was recorded. The results are shown in Fig. 7. While cellular synthesis (as opposed to energy generation and maintenance) mostly ceases during night, several synthesis fluxes can indeed be active during the night. In particular, the uptake of inorganic ions (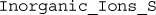) proceeds at a constant rate during the night. Likewise, ribosome synthesis, as well as lipid and DNA synthesis can proceed in the absence of light within the given range of kcat_*i*,*j*_. The catalytic efficiencies kcat_*i*,*j*_ that exert most influence on the night activities of synthesis reactions are those of ribosomes, those of ribosome synthesizing compounds, as well as those of amino acid synthesizing compounds. We note that the dependency of night activity upon the kcat_*i*,*j*_ is not necessarily confined to one-sided intervals. That is, for some compounds, night activity occurs for increase as well as decrease of kcat_*i*,*j*_. The latter finding indicates that compound synthesis during night is indeed the outcome of a global resource allocation problem and dependent on several factors, such as catalytic efficiencies, costs for storage and energy generation and competition between common building blocks, such as amino acids.

## Discussion

A computational analysis of phototrophic growth using constraint-based optimization requires to go beyond current time-invariant implementations of FBA. In particular, to describe the growth of cyanobacteria in a periodic environment necessitates new approaches that enable us to incorporate the temporal organization and conditional dependencies into a constraint-based description of metabolism. In this work, we have employed such an approach to describe the metabolic activity of the cyanobacteria *Synechocystis* sp. PCC 6803 over a full diurnal cycle. Based upon existing computational concepts^21^,^22^,^25^, we have incorporated conditional dependencies within cellular metabolism into a constraint-based analysis of cyanobacterial growth. Our approach allows us to simulate phototrophic growth of an average cell as a function of time in an environment with varying light intensity.

Our approach results in a time-course for all involved reaction fluxes, as well as changes in biomass composition over a diurnal cycle. The results are in reasonable agreement with several known properties for the temporal organization of phototrophic metabolism. We observe exponential growth during light period and only minor metabolic activity during night. Glycogen is accumulated before darkness, as also observed experimentally for some cyanobacteria^26^. Despite the reduced metabolism in the absence of light, synthesis of a few compounds may also proceed during darkness. Overall, however, reliable quantitative experimental observations for a detailed direct comparison are still scarce. Most currently available data relates to time-dependent gene transcription, often revealing strong diurnal dynamics, see^27^,^28^ for an overview. However, such changes do usually not translate straightforwardly into corresponding protein or flux changes. Studies of the proteome are scarce and typically reveal far less diurnal variation^29^. 13C labeling experiments over a full diurnal cycle, as would be necessary to reveal the actual changes in resource and flux allocation, are as yet not available.

Nonetheless, our simulation framework already allows us to formulate several hypotheses with respect to the optimality of metabolism under diurnal light availability. For example, to what extent a compound is synthesized in the absence of light strongly depends not only on its own catalyst but also the catalytic efficiency of other pathways. This finding reflects the fact that synthesis at night makes use of common storage compounds, hence strong coupling between all pathways must be expected. While it is known that *Synechocystis* sp. PCC 6803 exhibits only reduced metabolic activity at night, synthesis of individual compounds as well as transport may still be active, as also indicated by global transcription data that exhibit few genes that peak at night, including genes related to transport^28^. A striking feature of the simulation is the highly intermittent synthesis rates observed for some reactions, most notably the synthesis of enzymes. This behaviour is not due to numerical instabilities, but reflects the optimal temporal pattern of enzyme synthesis. While transcriptional bursts are well known, the time-resolution of currently available transcription data, usually only 6–8 data points per diurnal period, does not allow determining whether such intermittent synthesis rates also occur *in vivo*.

Similar to conventional FBA, our computational framework can be extended into a multitude of further applications, such as *in-silico* testing of knockout mutants and the identification of essential genes under diurnal light availability. As yet, we consider the approach presented here primarily as a proof-of-concept for the application of FBA on diurnal cyanobacterial metabolism. Necessary improvements include a more refined distinction between balanced cellular compounds and those which are allowed to accumulate, corresponding concepts were developed recently in more detail^24^. Likewise, while the computational demand is significantly higher than for conventional FBA, in particular when making use of flux variability analysis, it is feasible to apply our concept on metabolic networks of increasing, up to genome-scale, size. We can estimate the approximate computational effort for application on large-scale models using extrapolation of results obtained from finer time-discretization. See section “Materials” for details. However, for large networks we expect that flux variability and multiple alternate solutions require further attention. Similar as in conventional FBA, the flux solutions must not necessarily be unique and we expect significant variability for larger models.

Of high relevance from a biological perspective is to incorporate further conditional dependencies into the computational description of phototrophic metabolism. While in this study, most key interdependencies of phototrophic growth have been considered, the list is far from complete. For example, high light intensities are known to have adverse consequences including oxidative stress and reducing the lifetimes of key proteins. We therefore expect that trade-offs between capacity and potential damage in changing light are among the important factors that further shape cyanobacterial metabolism.

Taken together, we envision our framework to be able to contribute novel insights to the expected evolutionary trade-offs in metabolic resource allocation. In this respect, we consider the present analysis a pilot study that demonstrates the feasibility of diurnal simulations of metabolism using constraint-based approaches. We expect that aspects of our method must be expanded upon before the solution of the computational resource allocation reflects actual resource allocation of phototrophic growth. Nonetheless, our simulation results already give rise to several testable features of temporal resource allocation that can be verified or refuted in future experiments. We expect that this and similar methods will become instrumental to analyze, and eventually understand, the principles that shape metabolic resource allocation in complex environments.

## Methods

### A minimal model of *Synechocystis* sp. PCC 6803

Simulations were performed using a simplified model of the cyanobacterium *Synechocystis* sp. PCC 6803. The model incorporates the main metabolic processes required to describe phototrophic growth in a diurnal environment. The cell harvests energy from incoming light using the photosynthetic apparatus (reactions 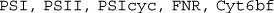 and 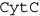) and produces cellular energy (ATP) and the reduction equivalent NADPH. At the oxygen-evolving complex of 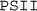 water is split and the resulting electrons reduce plastoquinone Q to QH_2_. The Cytb6f complex transfers the electrons to plastocyanin (PC). At PSI electrons are transferred to ferredoxin and further to NADPH via the ferredoxin-NADP+ reductase (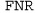). Cyclic electron flow is represented by a separate reaction 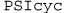. The electron transport chain (ETC) creates a proton gradient at the thylakoid membrane. This gradient is used by the ATPase to regenerate ATP.

To account for cellular growth, the model contains lumped overall reactions for the biomass components pigments, DNA, RNA, proteins, lipids, cell wall, amino acids, soluble pool and inorganic ions. These overall reactions produce the respective components out of 3-phosphoglycerate (C3), energy (ATP), redox (NADPH) equivalents, and inorganic compounds. The compound C3 is generated by the Calvin-Benson cycle. The cycle is split into the RubisCo reaction which fixes carbon dioxide and uses ribulose-1,5-bisphosphate (C5) as a precursor to generate two molecules of C3. The remaining part of the cycle is represented by an overall reaction which regenerates C5 from C3 (reaction 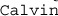).

The reactions 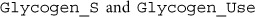 describe glycogen synthesis and mobilization, respectively. Glycogen is broken down via the TCA cycle to generate NADPH that serves as a substrate for respiration. The model is summarized in Table 1. All stoichiometries are derived from the genome-scale reconstruction^7^. The stoichiometries for the RNA and protein requirement, as well as for the ribosome synthesis were derived from the known ribosomal RNA content and the ribosomal protein content (KEGG) and their carbon content.

### Conditional dependencies and capacity limits

Rate dependencies are listed in Table 2. The level of Ribosomes limits the translational capacity. The level of 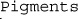 limits the light uptake and hence the activity of photosystems 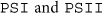. All metabolic reactions in the network have a catalyzing enzyme. Hence, these reactions are limited by the corresponding enzyme. The naming scheme of an enzyme for a specific reaction is the prefix 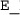 combined with the name of the reaction.

For many cellular compounds, precise estimates of their catalytic activities are not available. We therefore approximated the respective kcat_*i*,*j*_ values using a known static flux solution and available data on average compound abundances. Specifically, based on an average division time of approximately 24 h under diurnal illumination, we assume that the amount of cellular biomass components synthesized by the time-independent FBA model over a (light) period of 12 h is sufficient to catalyze its own flux distribution (self-consistency). To this end, we obtain estimated reference flux distributions for day and night conditions, denoted as 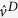 and 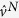 respectively, using conventional FBA with parameters as described previously^7^. Computational details are provided in the Supplementary Information. The integrated cellular components resulting from the conventional FBA solution are denoted as 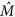. Formally, the capacity constraints for the capacity limiting compound *j* is given by


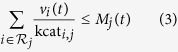


with the estimation


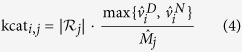


where 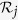 is the set of reactions catalyzed by the compound *j* and 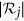 denoting the number of catalyzed reactions. In case a compound catalyzes several reactions, we assign equal amounts of the catalyzing compound to each reaction and estimate the kcat_*i*,*j*_ accordingly (with the exception of enzyme synthesis reactions, for which the ribosomes have the same catalytic efficiency). A complete list of dependencies is provided in Table 2.

### Reference compound amounts

The reference compound amounts 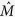 for the estimation of catalytic activities are obtained from the biomass composition of the genome scale model in^7^. In general they are identical to the stoichiometric coefficients in the biomass assembly reaction. However, the genome scale model only provides an estimate of total protein content, not the amount of individual enzymes allocated to synthesis pathways. Reference amounts for enzymes in the reduced model were therefore estimated using available proteomic data of *Synechococcus elongatus* PCC 7942^29^. The data contain protein counts per cell for 2178 proteins of 2854 proteins total. These data were converted to (relative) protein amounts assuming the measured proteins represent 100% of total proteins. Mapping of measured proteins to enzymes in the minimal model was performed according to the gene mappings provided by Beck *et al.*^30^. Importantly, most metabolic enzymes in the model represent lumped protein sets. The reference amount for each (lumped) protein set was obtained by summing over the respective set of (relative) protein abundance using gene annotation of the genome-scale model and GO annotation of the *Synechocystis* sp. PCC 6803 genes. We note that the lumped protein sets corresponding to lumped overall reactions are not disjoint. The reference amount of enzymes for the synthesis of the 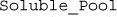 was assumed to be 1% of total proteins. The remaining proteins which could not be assigned to any lumped protein set were assigned to the pool of additional proteins 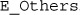. These proteins have no catalytic activity and must satisfy therefore a minimum quota. Furthermore the genome scale model does not explicitly contain ribosomes. Here the reference amount of ribosomes was estimated according to the quota of the ribosomal proteins. The reference amount for RNA was reduced by the ribosomal RNA amount correspondingly. Phosphate and water are explicitly included in the genome scale model but not in the presented model. The list of reference compound amounts is provided in the Supplementary Information.

### Additional constraints and dependencies

To represent overall cellular maintenance a Maintenance reaction is introduced that consists of two parts. First, there is a basal growth-independent minimal activity of the Maintenance reaction proportional to biomass. This proportion was chosen such that the Glycogen quota reported in a previous time-invariant FBA^7^ is completely utilized during night. Second, a minimal activity of the reaction Maintenance is coupled to the ATPase rate.

For the cellular components 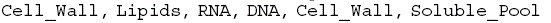 and 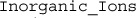 minimum quotas are defined. These constraints are required, since these components do not impose any capacity limit nor are they the educt of other reactions (excluding RNA which is required for the synthesis of ribosomes). The respective quotas are adopted from the biomass composition reported in Knoop et. al.^7^ We note that for some compounds, such as free amino-acids (AA) no lower bound was implemented, resulting in a nominal amount of zero during the simulation, despite non-zero flux through the compound. The results imply that free amino-acids are not accumulated, but the value zero should not be interpreted as an actual concentration or amount. The DNA quota is enforced only at the start, and hence implicitly at the end of the day, whereas other quotas have to be fulfilled at all times. This choice is motivated by the fact that, for example, 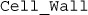 has to increase with system size, whereas DNA replication has only to be completed at certain time points.

### Conditional FBA

Similar to conventional FBA, our approach makes use of a stoichiometric matrix 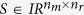 for *n*_*r*_ reactions and *n*_*m*_ compounds. In contrast to conventional implementations, only a subset 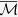 of compounds are assumed to be in steady state. Non-stationary compounds as well as all catalyzing compounds are represented by the set 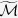. The subset of corresponding rows in the stoichiometric matrix are indicated by 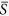 and 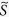 respectively.

The method starts with the initial amounts 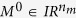 of the compounds in 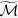. These amounts are not a priori chosen but are itself model variables. The method then estimates *n*_*t*_ flux distributions 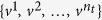 in *n*_*t*_ time intervals 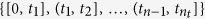. Compound amounts are updated at each time point *t*_*k*_ according to flux distribution *v*^*k*^ complying with the Euler scheme. The flux capacities in time interval [*t*_*k*_, *t*_*k*+1_] are linearly dependent on the compound amounts *M*^*k*^ at time point *t*_*k*_.

Additionally the method takes into account linear constraints on the compound amounts. The problem corresponds to a global optimization problem and is implemented as linear program (LP) as follows:


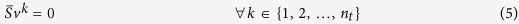



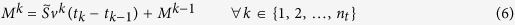



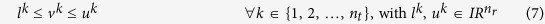



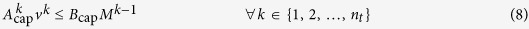



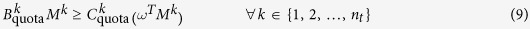



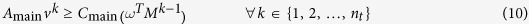



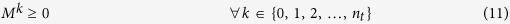



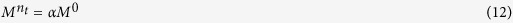



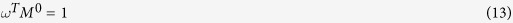


Constraint (5) enforces the mass balance for metabolites in steady state, constraint (6) updates the compound amounts at time point *t*_*k*_ according to the flux in time frame *k*, constraint (7) limits the capacities according to pre-assigned values and allows integration of environmental changes (when appropriate). The constraint (8) encodes the set of *n*_cap_ capacity constraints with matrices of inverse kcats 
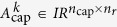
 and an index matrix 
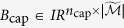
. We note that for the capacity constraints a separate 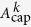 matrix is used for each *k*. While the kcat_*i*,*j*_ are assumed to be time-independent, the matrix 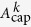 can be used to also specify time-dependent constraints. In our case, varying light intensity is implemented as a time-dependent effective kcat_*i*,*j*_ for the compound 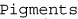 (see second line in Table 2). (9) is the set of quota constraints with index matrices 
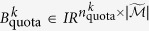
, quota matrices 
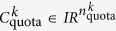
, the biomass weights 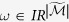 (*w*^*T*^ is the transposed vector) and the number of quota constraints 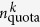 for each *k*. Constraint (10) encodes the set of *n*_main_ maintenance constraints with 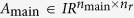 and 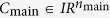. Constraints (8)–(10) are given in detail in Table 2. For different *k* the the quota constraint matrices 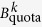 and 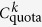 differ in the number of rows, because the DNA quota is enforced only at simulation start (and, implicitly, simulation end), whereas other quotas have to hold in each time point *t*_*k*_. Constraint (11) reflects the fact that compound amounts must not be negative. Constraint (12) enforces the synthesis of the compound vector (cyclic growth), with *α* denoting the fold change. Constraint (13) limits the starting amount of biomass. Biomass is defined as a weighted sum of all components in *M*^*k*^. The variables in the LP problem are *M*^0^, *v*^*k*^, and *M*^*k*^ for all *k* from 1 to *n*_*t*_.

The formulation allows for a global optimization for any given *α*. Our objective is the maximal biomass fold change *α*. The maximal value was estimated using bisection (see algorithm 1). The algorithm computes the maximal *α*, for which the LP has a solution.


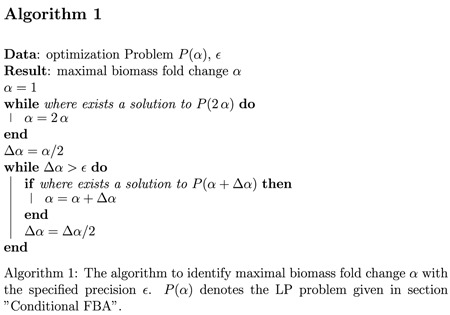


**Figure 8:**
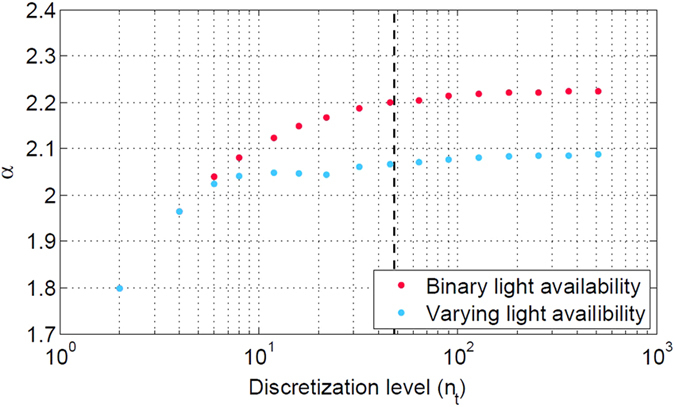
Dependency of maximal biomass fold change *α* on the number of intervals used for time discretization in the constraint optimization problem. The dashed line indicates the value used in the main text.

The solution is not necessarily unique. Variabilities of flux and compound levels are estimated as follows: Using the pre-determined (maximal) value of *α*, each flux value and each component level is minimized and maximized within each time interval. The difference between minimum and maximum is denoted as flux and compound level variability, respectively. We note that estimation of variability is computationally demanding and requires two optimization runs per flux/compound and per time interval. For the implementation *n*_*t*_ = 48 intervals were chosen, results for different choices of *n*_*t*_ are shown in Fig. 8. The results are robust with respect to the choice of *n*_*t*_. A run with a time-discretization of *n*_*t*_ = 96 is provided in the Supplementary Information to confirm robustness of the overall results.

### Implementation and runtime

The method was implemented in MATLAB^TM^ as a linear optimization problem. The optimizations were performed with Gurobi 12.2 on a 3.2 GHz computer (Intel® Xeon® Processor E5-1650) with 6 GB RAM. Simulations were tested with different solvers (Gurobi and CPLEX 12.6) with identical outcomes. The model size grows linearly with the number of compounds, the number of reactions, the number of additional constraints *n*_*c*_ and quadratically with the number of time steps 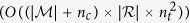. To check for the variabilities of the optimal solutions, two optimizations for each reaction and each time point had to be performed. All code is provided in the Supplementary Information. To estimate runtimes for large-scale models, we extrapolate results obtained from finer time discretization *n*_*t*_. For the presented model the runtime of one LP increased by a factor 10.000 with *n*_*t*_ increasing from 48 to 512 (about 10 min on the specified computer). The size of this problem would be equivalent to an LP for a model with 550 reactions, 550 compounds and *n*_*t*_ = 48.

## Additional Information

**How to cite this article**: Rügen, M. *et al.* Elucidating temporal resource allocation and diurnal dynamics in phototrophic metabolism using conditional FBA. *Sci. Rep.* **5**, 15247; doi: 10.1038/srep15247 (2015).

## Supplementary Material

Supplementary Information

Supplementary Information 1

## Figures and Tables

**Table 1:** Summary of model equations.

ATPase:	14 Hx + 3 ADP → 3 ATP
Calvin:	5 NADPH + 5 C3 + 8 ATP → 5 NADP + 3 C5 + 8 ADP
Cyt6bf:	QH2 + 2 oxPC → Q + 4 Hx + 2 redPC
CytC:	O2 + 4 redPC → 4 Hx + 4 oxPC
FNR:	2 redFd + NADP → 2 oxFd + NADPH
Maintenance:	ATP → ADP
NDH:	Q + NADPH → QH2 + 4 Hx + NADP
PSI:	photon + redPC + oxFd → oxPC + redFd
PSII:	4 photon + 2 Q → 2 QH2 + 4 Hx + O2
PSIcyc:	Q + 2 redFd → QH2 + 4 Hx + 2 oxFd
Rubisco:	C5 + CO2 → 2 C3
TCA:	4 NADP + C3 + ADP → 4 NADPH + ATP + 3 CO2
AA_S:	66.41 NADPH + 15.48 C3 + 19.89 ATP → 0.321 O2 + 66.41 NADP + 19.89 ADP + 1.483 CO2 + AA
Inorganic_Ion_S:	4.544 NADPH + 9.62 ATP → 4.544 NADP + 9.62 ADP + Inorganic_Ion
Cell_Wall_S:	49.46 NADPH + 14.82 C3 + 31.07 ATP + 0.4276 CO2 → 49.46 NADP + 31.07 ADP + Cell_Wall
DNA_S:	52.89 NADPH + 9.912 C3 + 40.27 ATP + 2.014 CO2 → 52.89 NADP + 40.27 ADP + DNA
Lipid_S:	61.82 NADPH + 18.48 C3 + 38.84 ATP → 2.678 O2 + 61.82 NADP + 38.84 ADP + Lipid
Pigment_S:	58.54 NADPH + 25.67 C3 + 21.73 ATP → 5.71 O2 + 58.54 NADP + 21.73 ADP + 12.54 CO2 + Pigment
RNA_S:	47.07 NADPH + 9.087 C3 + 38.54 ATP + 2.35 CO2 → 47.07 NADP + 38.54 ADP + RNA
Ribosome_S:	0.339 E_Ribosome_comp + 0.661 RNA + 0.21 ATP → Ribosome + 0.21 ADP
Soluble_Pool_S:	24.95 NADPH + 4.937 C3 + 15.69 ATP → 0.12 O2 + 24.95 NADP + 15.69 ADP + 1.716 CO2 + Soluble_Pool
Glycogen_S:	2 NADPH + 2 C3 + 2 ATP → 2 NADP + 2 ADP + Glycogen
Glycogen_Use:	2 NADP + Glycogen → 2 NADPH + 2 C3
Enzyme synthesis:	
*e*_S:	104.56 ATP + AA → 104.56 ADP + *e* 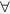 *e* ∈ {E_AA_S, E_ATPase, E_Inorganic_Ion_S, E_Calvin, E_Cell_Wall_S, E_Cyt6bf, E_CytC, E_DNA_S, E_FNR, E_Glycogen_S, E_Glycogen_Use, E_Lipid_S, E_Maintenance, E_NDH, E_Others, E_PSI, E_PSII, E_Pigment_S, E_RNA_S, E_Ribosome_S, E_Ribosome_comp, E_Rubisco, E_Soluble_Pool_S, E_TCA, E_Carbon_Uptake}
Exchange:	
CO2_Export:	CO2 →
O2_Export:	O2 ↔
Photon_Uptake:	→ photon
Uptake_Carbon:	ATP → ADP + CO2

Throughout the text, enzymes are denoted as E_<reaction name>. The naming scheme for enzyme synthesis reactions is <enzyme name>_S.

**Table 2:** Reaction constraints of conditional FBA.

Capacity constraints:	0.002549 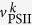 + 0.0006371 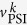 ≤ 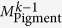
	*γ*^*k*^ 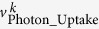 ≤ 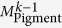
	4.36 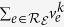 ≤ 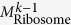
	0.3814 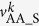 ≤ 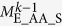
	0.001171 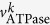 ≤ 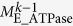
	12.88 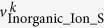 ≤ 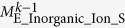
	0.008816 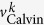 ≤ 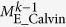
	0.4419 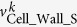 ≤ 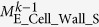
	0.00009621 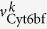 ≤ 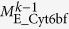
	0.000001396 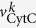 ≤ 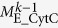
	2.515 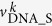 ≤ 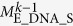
	0.0008281 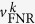 + 0.0008281 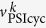 ≤ 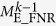
	0.001658 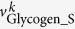 ≤ 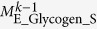
	0.0001272 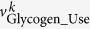 ≤ 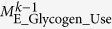
	17.49 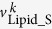 ≤ 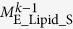
	0.00005342 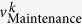 ≤ 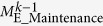
	0.00278 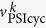 + 0.00278 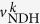 ≤ 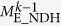
	0.001041 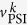 ≤ 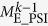
	0.02612 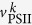 ≤ 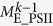
	3.423 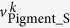 ≤ 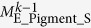
	1.084 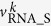 ≤ 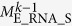
	0.2214 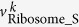 ≤ 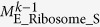
	0.002621 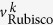 ≤ 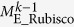
	3.193 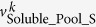 ≤ 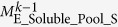
	0.0222 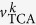 ≤ 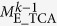
	0.0003567 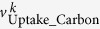 ≤ 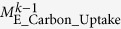
Quota constraints:	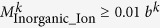
	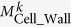 ≥ 0.059 *b*^*k*^
	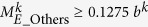
	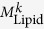 ≥ 0.12 *b*^*k*^
	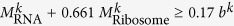
	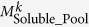 ≥ 0.029 *b*^*k*^
	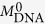 ≥ 0.031 *b*^0^
Maintenance constraints:	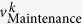 − 0.05 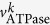 ≥ 0
	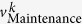 ≥ 0.00641 *b*^*k*^

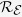
 is the set of enzyme synthesizing reactions. *b*^*k*^ is the amount of biomass at time point *t*^*k*^, which is a weighted sum of compounds (=*w*^*T*^*M*^*k*^). The term *γ*^*k*^ denotes the inverse of the time-dependent effective catalytic efficiency of light harvesting of pigments in the *k*-th interval (in the case of varying light). Coefficients in the capacity constraints have the units [s]. Coefficients in quota and maintenance constraints are dimensionless.
