# Supplementary material for: Elucidating temporal resource allocation and diurnal dynamics in phototrophic metabolism using conditional FBA
Source: Sci Rep. 2015 Oct 26;5:15247. doi: 10.1038/srep15247 (PMC4620596; doi:10.1038/srep15247)
Supplement: Supplementary Information [file srep15247-s1.pdf]

Supplementary Information

Elucidating temporal resource allocation and diurnal dynamics in phototrophic metabolism using conditional FBA

Marco Rügen*[1,2], Alexander Bockmayr[2], and Ralf Steuer†[1]

[1]Humboldt-Universität zu Berlin, Institut für Theoretische Biologie (ITB), Invalidenstr. 43, D-10115 Berlin, Germany
[2]Freie Universität Berlin, Research Center Matheon, FB Mathematik und Informatik, Arnimallee 6, D-14195 Berlin, Germany

August 15, 2015

The reference flux distribution

The day and night reference flux distributions $\hat{v}^D$ and $\hat{v}^N$ were computed by standard time-invariant FBA with a light intensity that results in approximately one doubling per day (12h light phase). At dawn the system is assumed to start with the reference compound levels in $\widehat{M}$ (note that $\widehat{M}$ is fixed) and to grow exponentially at a steady rate such that after 12 hours the compounds in $\widehat{M}$ are again produced (cell doubling). So we end up with 2 $\widehat{M}$ before light is switched off. The only exception is Glycogen. The assumption here is that we start with no Glycogen ($\neq \widehat{M}_{\mathsf{Glycogen}}$) and end up with 2 $\widehat{M}_{\mathsf{Glycogen}}$ before the darkness. So 2 $\widehat{M}_{\mathsf{Glycogen}}$ of Glycogen have to be produced during the first 12 hours.
During night the cell is assumed to perform maintenance (ATPase) only.

Estimation of v^N and v^D

The stoichiometric matrix S was extended to S^* by adding a biomass assembly reaction, the ATPase dependent Maintenance constraint and a Glycogen import reaction. One mmol biomass in the assembly reaction is formed by the compounds given in $\widehat{M}$ (estimated compound quotas), but with twice of Glycogen.
v^N was determined by solving the following LP:

$$\begin{aligned} \arg\min_{v^{N*}} \quad & v^{N*}_{\text{Glycogen\_Import}} \\ \text{s.t.} \quad & S^* v^{N*} = 0 \\ & v^{N*}_{\text{Photon\_Uptake}} = 0 \\ & v^{N*}_{\text{Maintenance}} = 0.00641 ||\widehat{M}||_1 \end{aligned}$$

v^N is v^{N*} without the components representing columns added in S^*. $||\widehat{M}||_1$ is indicating the sum of the elements in $\widehat{M}$.

*marco.ruegen@fu-berlin.de
†ralf.steuer@hu-berlin.de

v^D was determined by solving the following LP:

$$\begin{aligned} \underset{v^{D*}}{\arg\min} \quad & v^{D*}_{\text{Photon\_Uptake}} \\ \text{s.t.} \quad & S^* v^{D*} = 0 \\ & v^{D*}_{\text{Biomass\_production}} = \frac{ln2}{12}(||\widehat{M}||_1 + \widehat{M}_{\text{Glycogen}}) \\ & v^{D*}_{\text{PSIcyc}} = 0 \\ & v^{D*}_{\text{Glycogen\_Import}} = 0 \end{aligned}$$

v^D is v^{D*} without the components representing columns added in S^*.

For both optimizations the variability of the solution was analyzed.

Biomass weights w^T

Glycogen has the weight 0.2216. All other compounds have the weight 1.

Compound quotas & amounts

		Simulated compound amounts in					
		binary light condition			varying light condition		
compound i	$\widehat{M}_i$	min	mean	max	min	mean	max
E_Others	0.13	0.13	0.13	0.13	0.12	0.12	0.13
E_PSII	0.13	0.11	0.11	0.12	0.095	0.1	0.12
Ribosome	0.12	0.13	0.16	0.17	0.12	0.15	0.18
Lipid	0.12	0.12	0.12	0.12	0.11	0.12	0.12
E_Lipid_S	0.12	0.13	0.14	0.16	0.1	0.12	0.14
RNA	0.089	0.056	0.065	0.082	0.05	0.066	0.084
Cell_Wall	0.059	0.059	0.059	0.06	0.056	0.057	0.059
Glycogen	0.047	0	0.033	0.12	0	0.04	0.15
DNA	0.031	0.022	0.025	0.031	0.019	0.024	0.03
Soluble_Pool	0.029	0.029	0.029	0.029	0.027	0.028	0.029
Pigment	0.024	0.026	0.026	0.027	0.036	0.039	0.041
E_PSI	0.02	0.023	0.024	0.025	0.02	0.022	0.025
E_AA_S	0.011	0.012	0.015	0.016	0.011	0.012	0.015
E_RNA_S	0.01	0.012	0.012	0.015	0.0099	0.011	0.013
Inorganic_Ion	0.01	0.01	0.013	0.016	0.0095	0.012	0.015
E_FNR	0.0079	0.0092	0.0095	0.01	0.008	0.0086	0.0099
E_Inorganic_Ion_S	0.0071	0.0033	0.0054	0.0066	0.0029	0.0046	0.0058
E_Calvin	0.007	0.0082	0.0085	0.0091	0.0073	0.0077	0.0086
E_NDH	0.0068	0.0079	0.0084	0.0088	0.0064	0.0072	0.0088
E_Rubisco	0.0063	0.0073	0.0076	0.0081	0.0065	0.0069	0.0076
E_ATPase	0.0052	0.0061	0.0063	0.0066	0.0052	0.0057	0.0065
E_Soluble_Pool_S	0.0051	0.0053	0.006	0.0073	0.0045	0.0052	0.0064
E_Pigment_S	0.0046	0.005	0.0055	0.0069	0.0073	0.01	0.014
E_DNA_S	0.0043	0.0021	0.003	0.0043	0.0013	0.002	0.0027
E_Ribosome_S	0.0015	0.0013	0.0016	0.0028	0.001	0.0013	0.0022
E_Cell_Wall_S	0.0014	0.0017	0.0018	0.002	0.0016	0.0017	0.0021
E_Cyt6bf	0.00092	0.0011	0.0011	0.0012	0.00092	0.001	0.0012
E_Carbon_Uptake	0.00084	0.00097	0.001	0.0011	0.00086	0.00092	0.001
E_TCA	0.0008	0.00011	0.0002	0.00024	0.00014	0.00025	0.00031
E_Glycogen_S	0.00034	6e-05	0.0001	0.00012	0.00014	0.00024	0.00029
E_Glycogen_Use	2.9e-05	6e-07	1.1e-06	1.3e-06	8e-07	1.4e-06	1.7e-06
E_Maintenance	1.2e-05	1.4e-05	1.4e-05	1.5e-05	1.2e-05	1.3e-05	1.5e-05
E_CytC	1.7e-06	1.3e-08	2.4e-08	2.8e-08	6.5e-09	1.2e-08	1.4e-08
AA	0	0	9.7e-06	0.00019	0	0.00044	0.0076
E_Ribosome_comp	0	0	0.003	0.013	0	0.0057	0.019

Table 1: Estimated compound amounts and simulated compound amounts. $\widehat{M}_i$ represents the reference amount for compound i. Note that reference amounts are used to obtain estimates for k_{cat} but are not itself used in the simulations (except to specify a minimal quota as described in the main text).

Capacity utilization

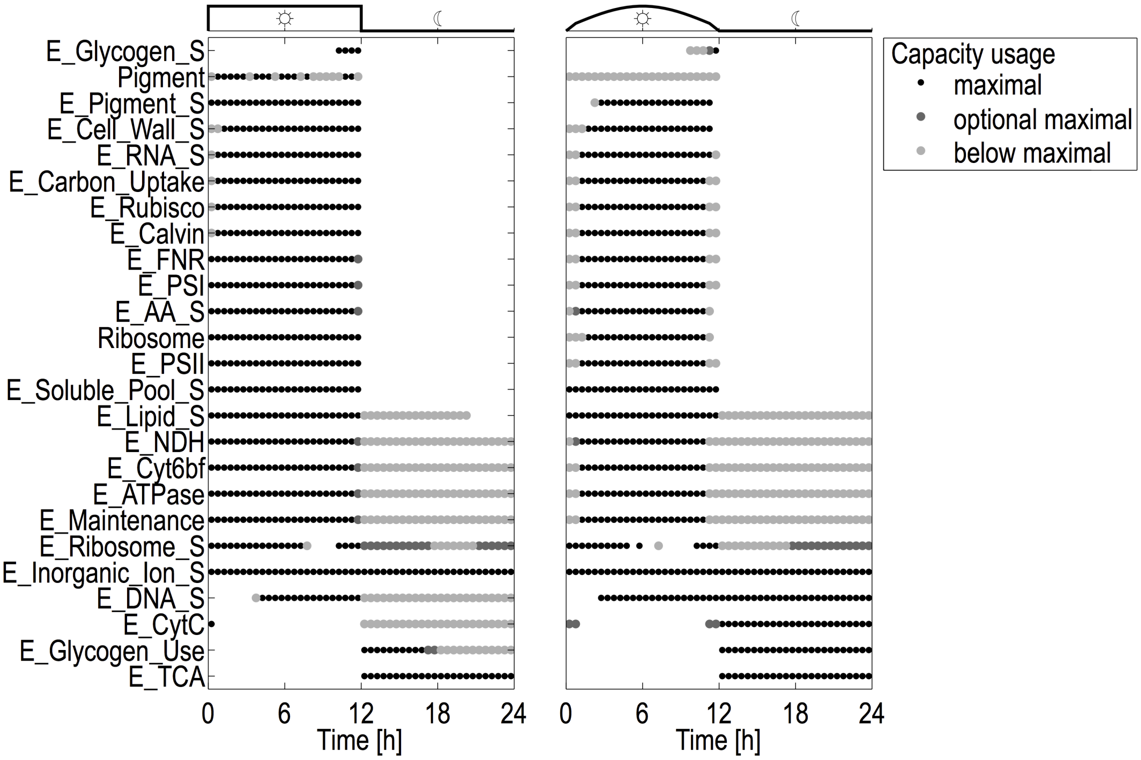

Figure 1: Capacity utilization over a diurnal cycle. Shown is the capacity usage of synthesis fluxes over a full diurnal cycle. The left panel shows the scenario where light uptake is constraint by the amount of pigments only, the right panel shows the scenario using a bell-shaped light availability. The symbol (•) indicates that the synthesis reaction operates at maximal capacity, that is, it equals kcat multiplied with the amount of the catalyzing compound. The symbol (•) indicates that the synthesis reaction may operate at maximal capacity but flux variability also allows for solutions where the flux is less than maximal capacity. The symbol •) indicates that the synthesis flux operates below maximal capacity. For a bell shaped light curve, pronounced regions of synthesis fluxes below maximal capacity exist, in particular during early morning and late afternoon.

Detailed simulation results for $n_t = 48$

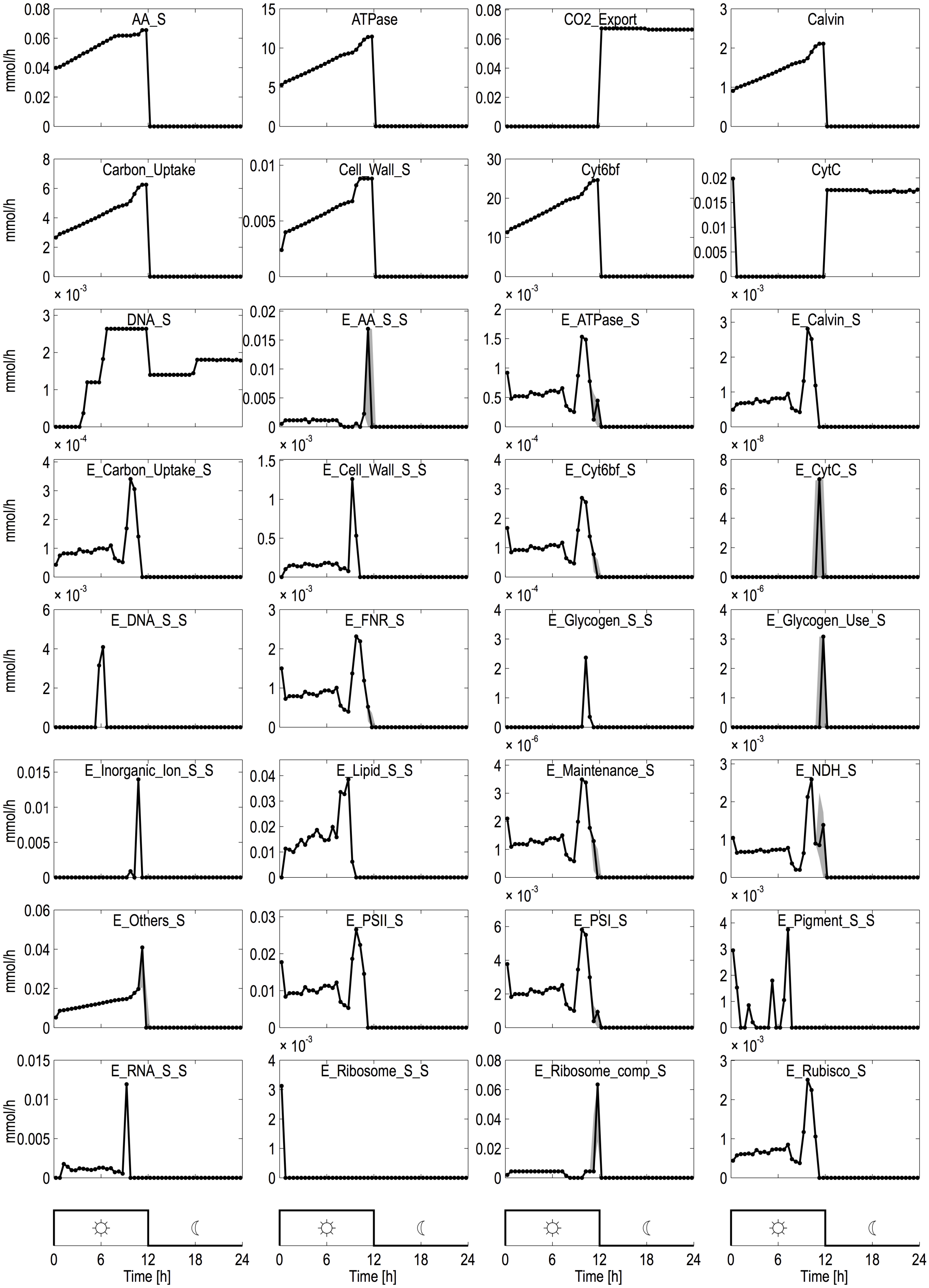

Figure 2: Complete set of flux rates and compound amounts over a full diurnal cycle (light uptake only limited by pigments). Shaded areas denote flux variability.

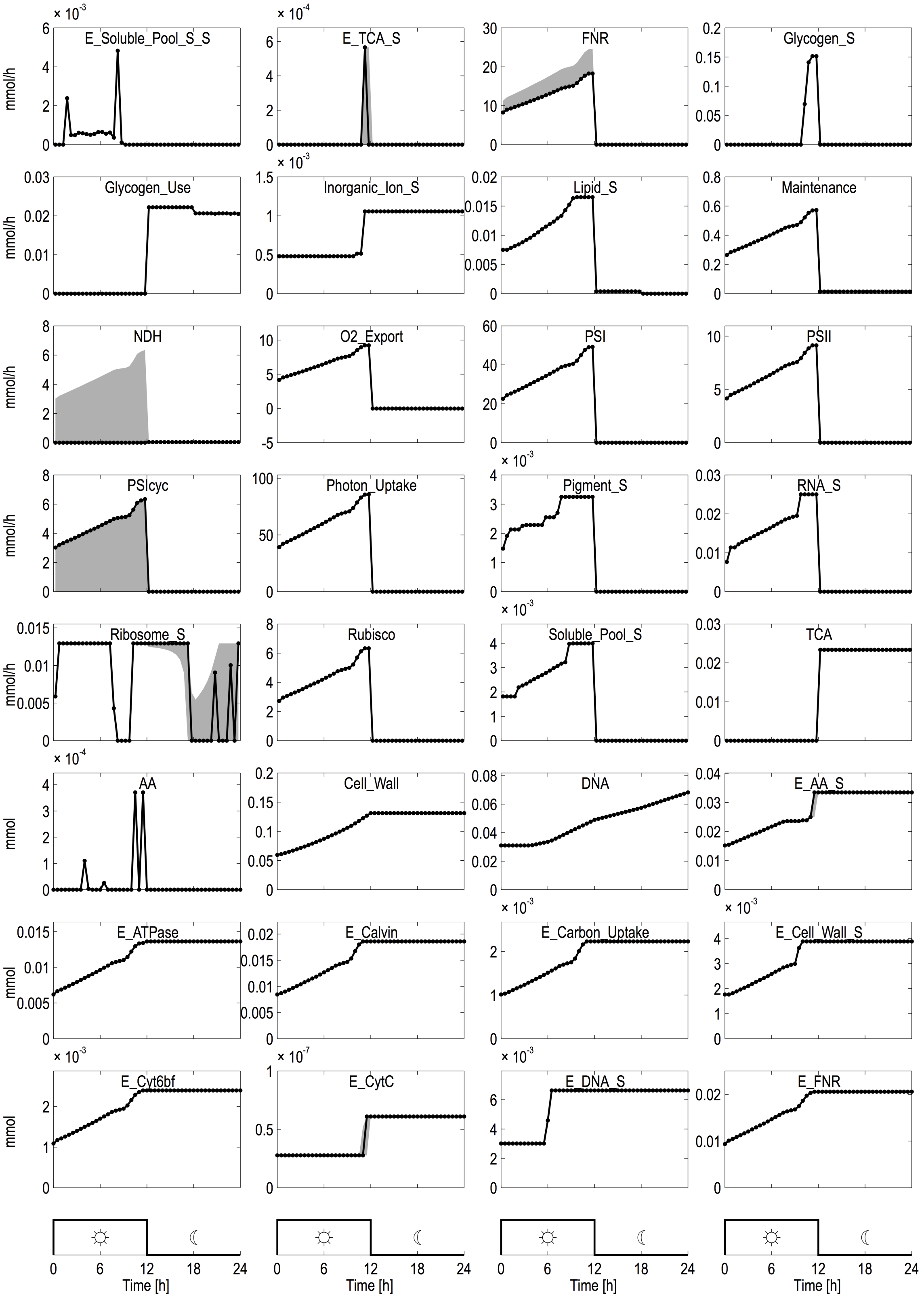

Figure 3: Continued: Complete set of flux rates and compound amounts over a full diurnal cycle (light uptake only limited by pigments). Shaded areas denote flux variability.

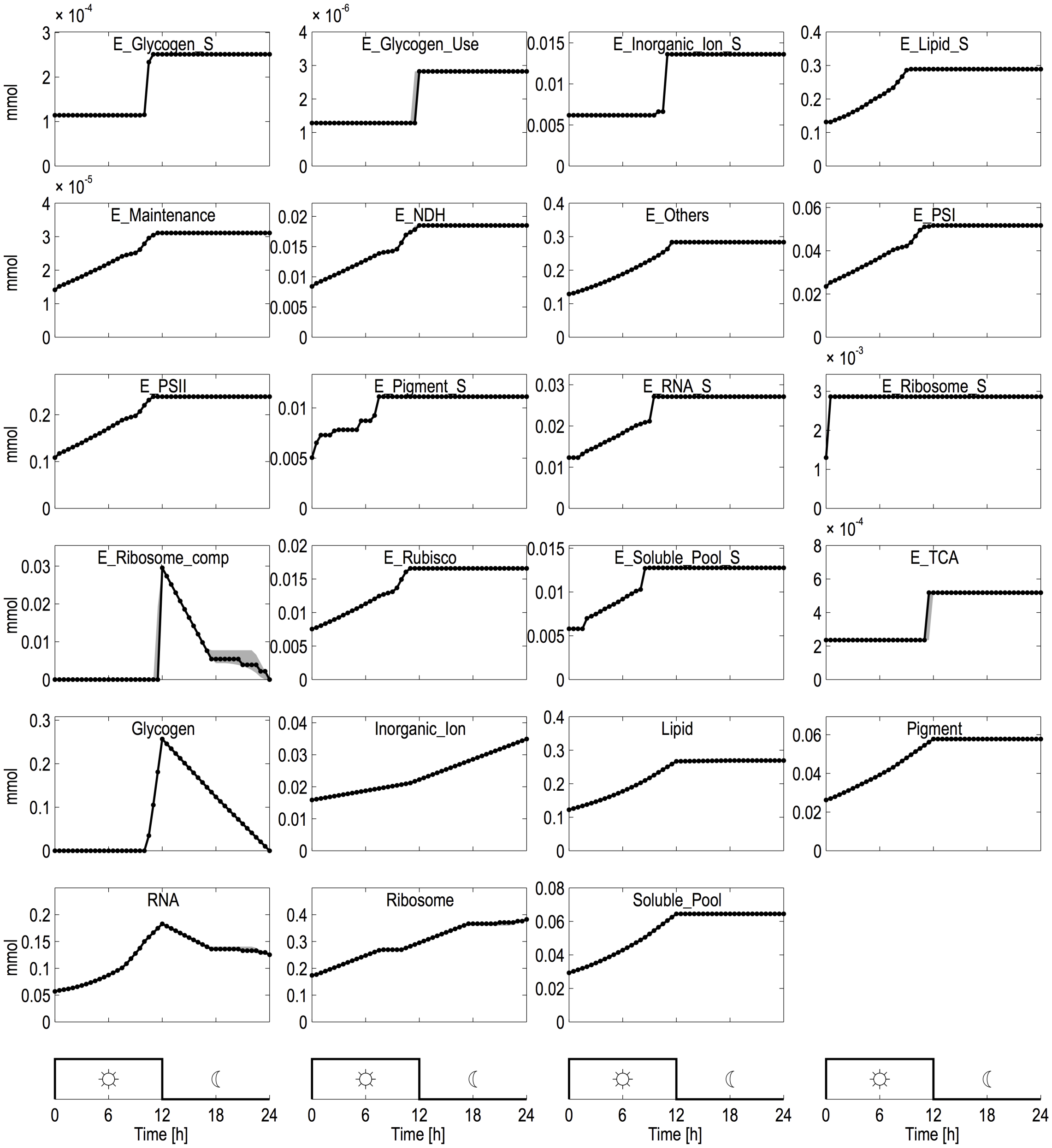

Figure 4: Continued: Complete set of flux rates and compound amounts over a full diurnal cycle (light uptake only limited by pigments). Shaded areas denote flux variability.

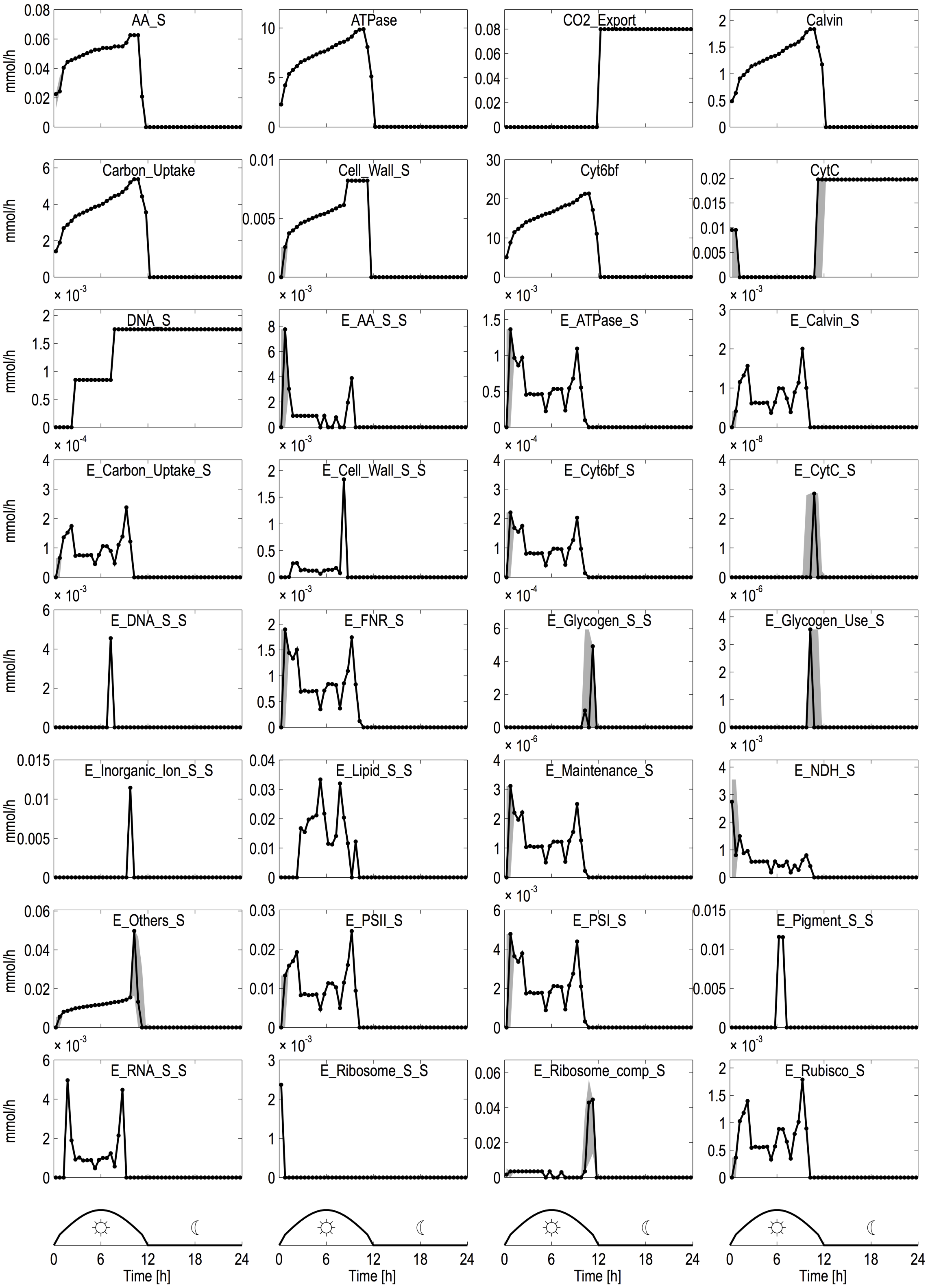

Figure 5: Complete set of flux rates and compound amounts over a full diurnal cycle (bell-shaped light availability). Shaded areas denote flux variability.

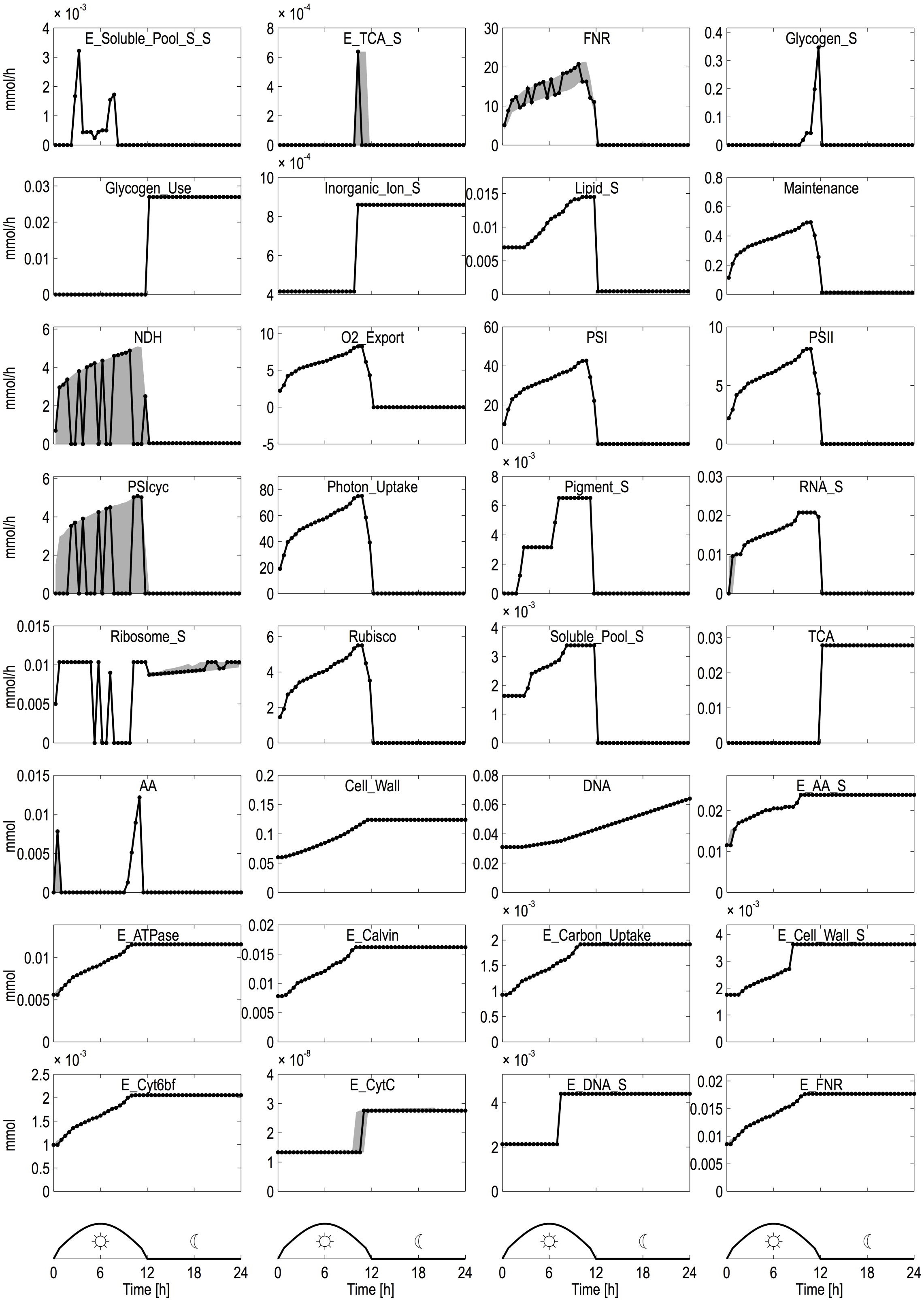

Figure 6: Continued: Complete set of flux rates and compound amounts over a full diurnal cycle (bell-shaped light availability). Shaded areas denote flux variability.

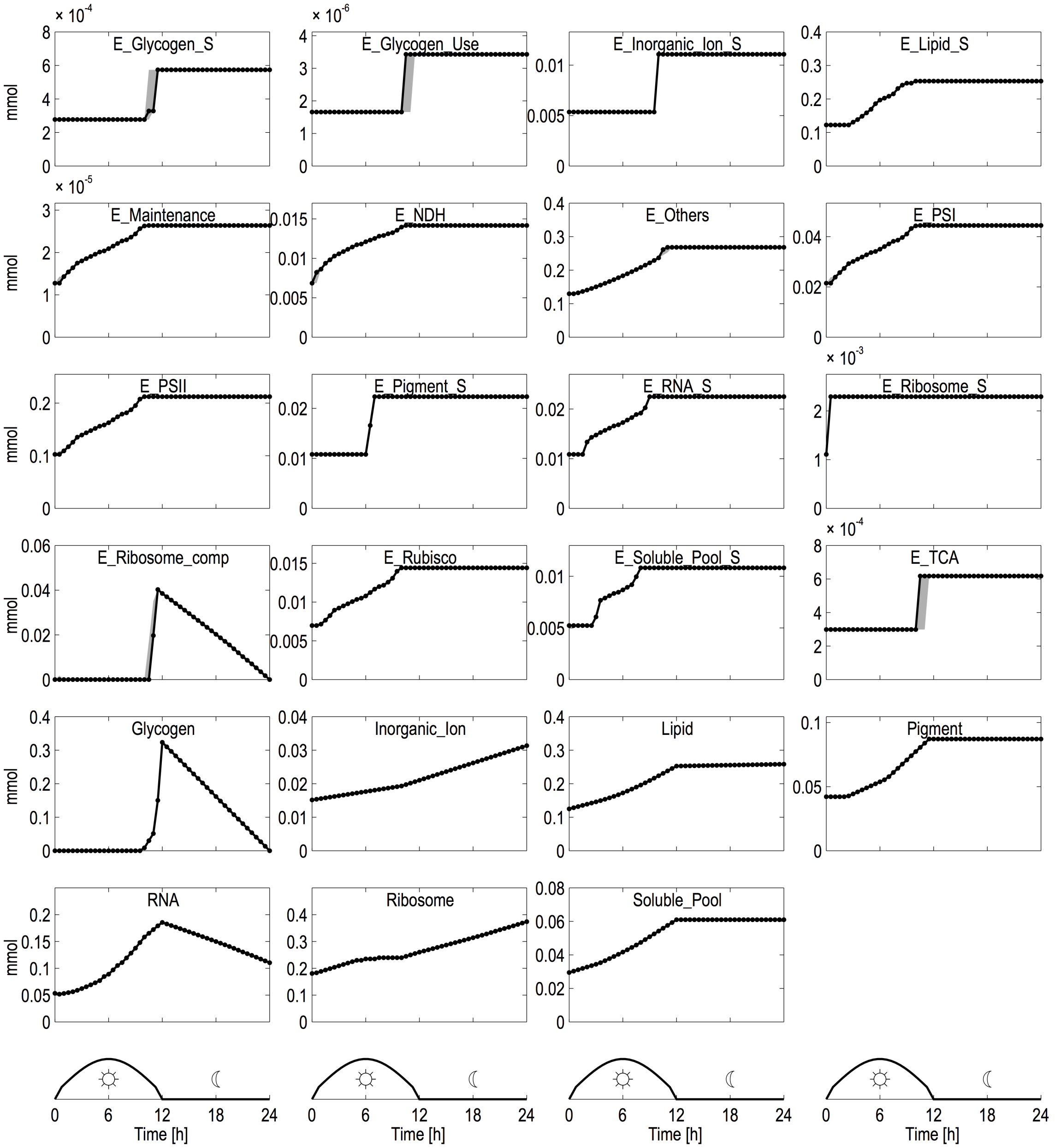

Figure 7: Continued: Complete set of flux rates and compound amounts over a full diurnal cycle (bell-shaped light availability). Shaded areas denote flux variability.

Simulation results for $n_t = 96$

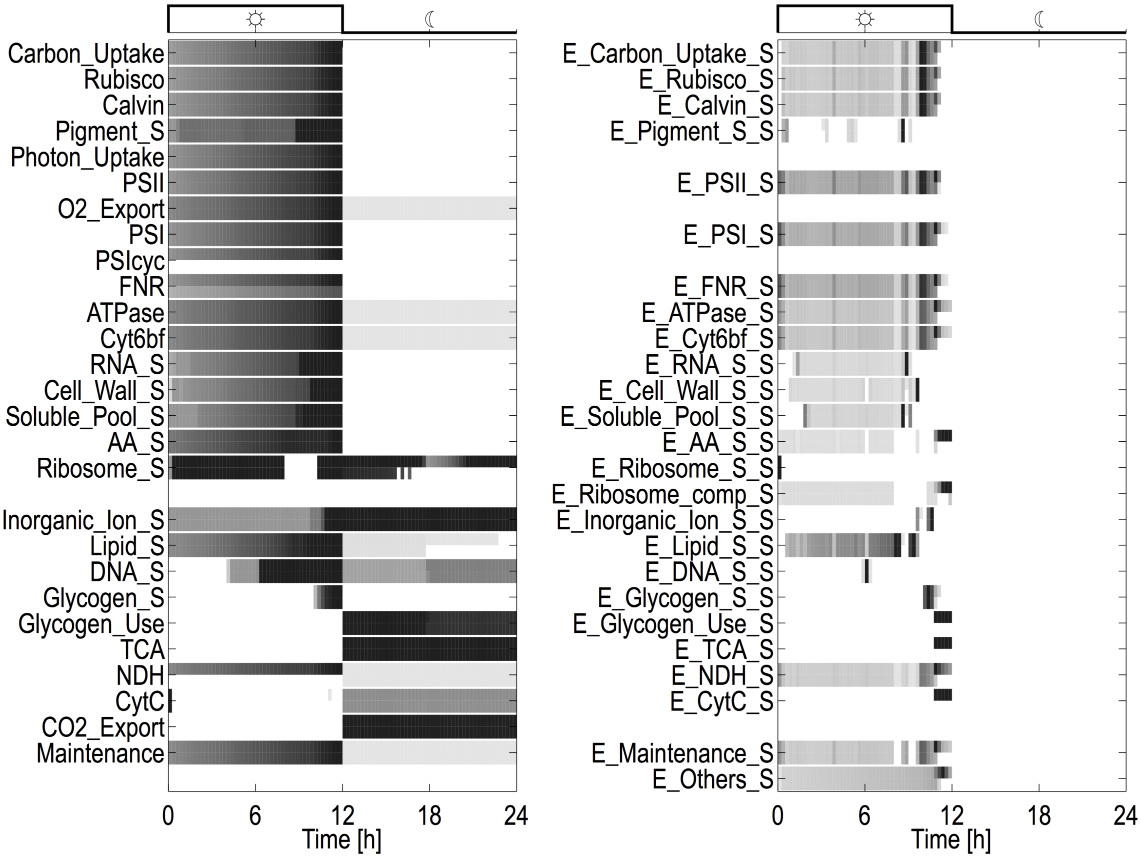

Figure 8: Flux rates over a full diurnal cycle and a time discretization of $n_t = 96$ steps. A prefix `E_` denotes compounds that catalyze reactions. A suffix `_S` denotes the synthesis flux of the respective biomass compounds. For example, the term `Carbon_Uptake` denotes the flux through the carbon uptake reaction, while `E_Carbon_Uptake_S` denotes the rate at which the transporter is synthesized. Flux rates are normalized such that the color ■ indicates the maximum value. White color indicates no flux activity. Each rate is represented by two rows. The upper row encodes the upper bounds of the normalized flux rates, the lower rows encode lower bounds. In the absence of flux variability, both rows coincide. The flux `O2_Export` switches sign during the cycle, with negative rates during night (net O_2 uptake). Shown is the absolute value.

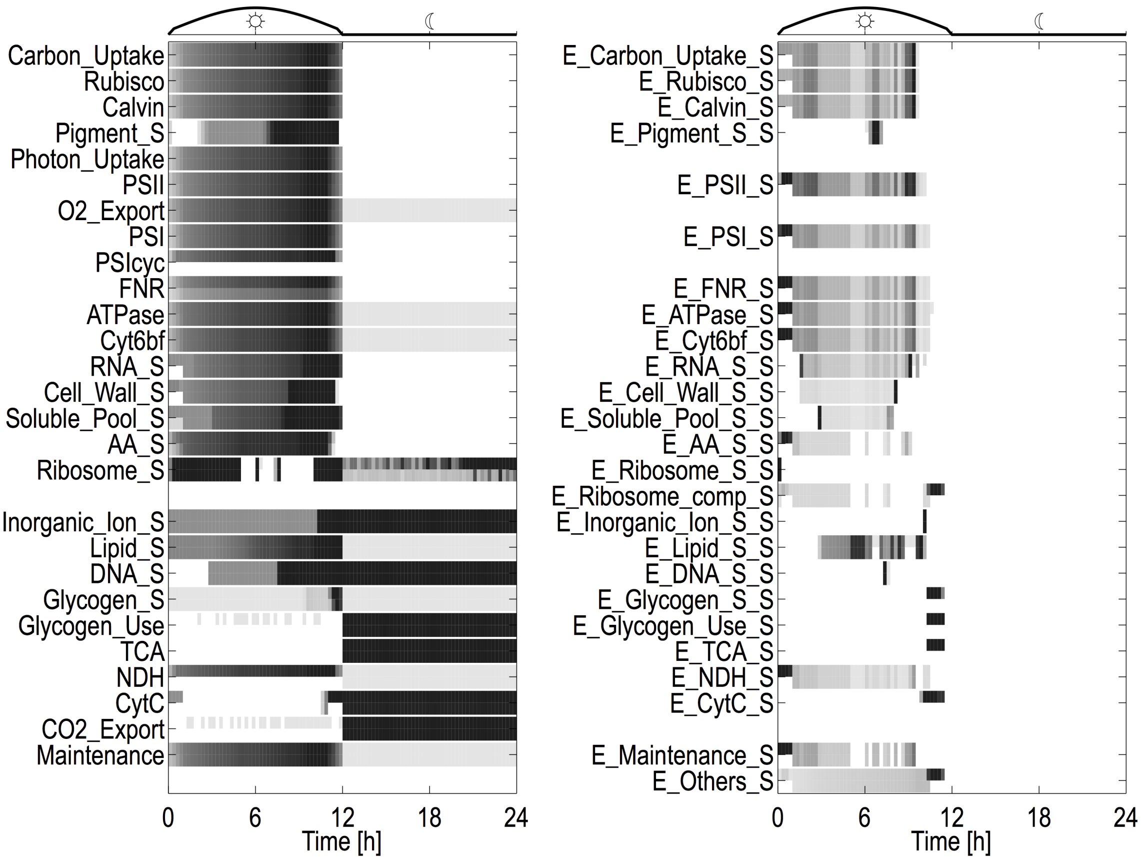

Figure 9: Flux rates over a full diurnal cycle using a bell shaped light curve and a time discretization of $n_t = 96$ steps. A prefix `E` denotes compounds that catalyze reactions. A suffix `S` denotes the synthesis flux of the respective biomass compound. Flux rates are normalized such that the color ■ indicates the maximum. White color indicates no flux activity. Each rate is represented by two rows. The upper row encodes the upper bounds of the normalized flux rates, the lower rows encode lower bounds. In the absence of flux variability, both rows coincide. The flux `O2_Export` switches sign during the cycle, with negative rates during night (net O_2 uptake). Shown is the absolute value.

References

1. Knoop, H. *et al.* Flux balance analysis of cyanobacterial metabolism: the metabolic network of Synechocystis sp. PCC 6803. *PLoS computational biology* **9**, e1003081+ DOI:10.1371/journal.pcbi.1003081 (2013).
