# Supplementary material for: Elucidating temporal resource allocation and diurnal dynamics in phototrophic metabolism using conditional FBA
Source: Sci Rep. 2015 Oct 26;5:15247. doi: 10.1038/srep15247 (PMC4620596; doi:10.1038/srep15247)
Supplement: Supplementary Information 1 [file srep15247-s2.zip › Manual.pdf]

Supplemental Information
Code Manual

Elucidating temporal resource allocation and diurnal dynamics in phototrophic metabolism using conditional FBA

Marco Rügen*[1,2], Alexander Bockmayr[2], and Ralf Steuer†[1]

[1]Humboldt-Universitt zu Berlin, Institut fr Theoretische Biologie (ITB), Invalidenstr. 43, D-10115 Berlin, Germany
[2]Freie Universität Berlin, Research Center Matheon, FB Mathematik und Informatik, Arnimallee 6, D-14195 Berlin, Germany

July 8, 2015

Quick start

To start the simulation of the simple example model given in figure 1, run the script `start_Demo()`.

To start the simulation of the reduced *Synechocystis* sp. PCC 6803 model:

1. load either the model with binary light availability or the model with varying light availability by:
   `load models/SynechocystisReduced_BinaryLight.mat`
   or
   `load models/SynechocystisReduced_VaryingLight.mat`
2. run simulation:
   `run_cFBA(model)`

Note: To view the results of *Synechocystis* simulation, load the result data and use the methods '`plotFlux`' or '`plotCompound`' as described in section 'Methods'.

Installation instructions

The code requires MATLAB and CPLEX. It was tested with MATLAB version 7.12 and CPLEX version 12.6. The CPLEX-MATLAB interface must be installed and its folder must be in the MATLAB path.
Several functions require to create some folder in the script folder, e.g. a 'results' folder to store the optimization results into.
No further installation required.

Test your environment by running the script `start_Demo()`

*marco.ruegen@fu-berlin.de
†ralf.steuer@hu-berlin.de

Code Information

Methods

Main methods:

- `start_Demo`
  Loads the simple example model from figure 1, runs the simulation and shows the results.

- `run_cFBA`
  Starts the simulation for a specified model and store the results in the folder '`results`'.
  Example call: `run_cFBA( model );`

- `plotCompound`
  Shows the simulated compound amounts of a specified simulation result and a specified compound.
  Example call: `plotCompound( res, 'Glycogen' );`

- `plotFlux`
  Shows the simulated flux rates of a specified simulation result and a specified reaction.
  Example call: `plotFlux( res, 'ATPase' );`

Methods called by the main methods:

- `create_cFBAProblem_FromModel`
  Create the LP problem for a specified model.

- `getCompoundStartAmountVariabilities`
  Computes the variabilities of the compound amount at simulation start for the specified problem.

- `getCompoundVariabilities`
  Computes the variabilities of the compound amount at all other time points for the specified problem.

- `getFluxVariablities`
  Computes the variabilities of all flux rates for the specified problem.

- `lp_solve`
  Calls the solver of interests.

- `setMaximalCompoundFoldChange`
  Computes the maximal compound fold change for the specified model.

- `setCompoundFoldChange`
  Sets the compound fold change of the specified LP problem to the a specified value.

- `time`
  Returns a stamp of the current time.

Data structures

- `model`
  Input model that contains the fields:

`rxns`	List of reactions
`mets`	List of compounds
`S`	Stoichiometric matrix S.
`lb`	Static lower flux bounds l^k for all intervals k.
`lb_var`	Optional. Structure for lower flux bounds specific for each interval. The fields are names of the reactions for which such varying lower bounds are defined. The fields are arrays with n_t elements. The elements define a bound for each interval. Overrides `lb`.
`ub`	Static upper flux bounds u^k for all intervals k.
`ub_var`	Optional. Structure for upper flux bounds specific for each interval. The fields are names of the reactions for which such varying upper bounds are defined. The fields are arrays with n_t elements. The elements define a bound for each interval. Overrides `ub`.
`rev`	Array of boolean values indicating, whether a reaction is reversible (true=reversible).
`ImbalancedMets`	Array of indices of imbalanced compounds.
`Biomass`	Array of compounds weights ($= w$).
`Constraints`	Constraint structure with fields:
`Capacities`	Optional. Capacity constraints. Structure with fields:
`A`	Part of A^k_{cap} which is identical for all k.
`B`	Part of B^k_{cap} which is identical for all k.
`gamma`	Optional. Array of γ^k values for varying light.
`Quotas`	Optional. Quota constraints. Structure with fields:
`ForAllTimePoints`	Optional. Quota constraints which are identical for all k. Structure with fields:
`B`	Part of B^k_{quota} which is identical for all k.
`C`	Part of C^k_{quota} which is identical for all k.
`ForStart`	Optional. Quota constraints for simulation start ($k = 0$). Structure with fields:
`B`	Part of B^k_{quota} which is defined only for $k = 0$.
`C`	Part of C^k_{quota} which is defined only for k.
`Maintenance`	Maintenance constraints. Structure with fields:
`A`	A_{main}
`C`	C_{main}
`dT`	Array of interval lengths.
`nT`	Number intervals.
`T`	Simulation time.
`ID`	Identifier string of the model.

- `res`
  Results structure that contains the fields:

`Prob`	LP problem structure with fields:
`S`	All constraints of the LP problem in one matrix.
`b`	Right hand side of the constraints.
`lecon`	Array of boolean values indicating whether the row constraints in `S` are $\leq$ or $=$ the corresponding element in `b`.
`lb`	Lower bounds of the variables in the LP problem.
`ub`	Upper bounds of the variables in the LP problem.
	Note: If x are the variables in the LP problem, then `lb`$\leq x \leq$`ub` and `S`$x \leq / =$`b` have to hold ($\leq / =$ means $\leq$ or $=$ depending on `lecon`).
`Vars`	Structure for indices of variables with fields:
`Fluxes`	Structure-Array for indices of flux variables with fields:
`Indices`	Array of indices of flux variables for a specific interval.
`AbsFluxes`	Structure-Array for indices of absolute flux variables with fields:
`Indices`	Array of indices of absolute flux variables for a specific interval.
`ImbMets`	Structure for compound amounts with fields:
`StartAmounts`	Array of indices of compound start amounts.
`S`	Matrix of linear combinations for metabolite amounts.
`Tind`	Array of indices of last columns in `S` to consider.
`Cons`	Structure for constraint indices with fields:
`CompoundFoldChange`	Array of indices of the fold change constraint.
`model`	Input model
`CompoundFoldChange`	Compound fold change ($= \alpha$)
`x0`	A solution to the LP.

Folders

- `models`
  Contains the reduced *Synechocystis* sp. PCC 6803 models in *.mat files. The model format is described in section 'Data structures'.

- `results`
  Contains the simulation results in `*.mat` files. The format is described in section 'Data structures'. The folder is initially empty. The method '`run_cFBA`' stores the result automatically in this folder.

- `log`
  This folder is initially missing. If logging shall be enabled, this folder has to be created.

- `tmp`
  This folder is initially missing and is created if the simulation runs into problems.
